# Development of the coupled smoothing technique *λ*S-FEM for mechanical analysis of twist drills

**DOI:** 10.1038/s41598-025-03520-8

**Published:** 2025-07-01

**Authors:** Qiuxia Fan, Qianqian Hua, Jianyu Li, Weihuang Liu, Qianqian Zhang, Zhuang Wen, Chan Xu, Lei Xu

**Affiliations:** School of Automation and Software Engineering, Shanxi University, Taiyuan, 030006 Shanxi China

**Keywords:** S-FEM, Scale factor, Gradient smoothing technique, Coupled, Twist drill, Solid mechanics, Applied mathematics, Mechanical engineering

## Abstract

A coupled smoothing technique, *λ*S-FEM, is introduced to improve the accuracy of numerical simulations in the mechanical analysis of twist drills. This method combines the edge-based smoothing finite element method (ES-FEM) with the node-based smoothing finite element method (NS-FEM). The *λ*S-FEM model is designed to evaluate the mechanical properties of twist drills made from tungsten carbide (WC), titanium nitride (TiN) coatings, and high-speed steel (M35), providing a theoretical basis for lifespan estimation and wear prediction. Linear tetrahedral elements construct the smoothing domain, and optimized weighting parameters balance and combine the smoothed strains from ES-FEM and NS-FEM. This integration enhances the accuracy of solutions for displacements, stresses, and strain energies, constructing stiffness matrices with optimal precision. The method’s feasibility is demonstrated through numerical case studies involving flange and shell extractor components. Analyses of straight shank twist drills compare displacement and stress magnitudes across FEM, S-FEM, and *λ*S-FEM under various degrees of freedom (DOF). Results show *λ*S-FEM significantly reduces errors, particularly with coarse meshes, validating its practical application in solving engineering challenges.

## Introduction

The twist drill, a crucial tool in drilling operations, is valued for its simple design, ease of use, adaptability, and cost-effective ability to perform high-precision machining. It is widely employed for processing materials such as metal, wood, and stainless steel. However, despite its advantages, twist drills are prone to wear and deformation during machining. These challenges have motivated extensive research into the stress, wear, and deformation characteristics of twist drills. Dewangan et al.^1^ analyzed the stress and deformation of WC-Co twist drills under granite conditions, identifying critical wear phenomena. Similarly, Prałat et al.^2^ explored the relationship between drill bit hardness and wear resistance, demonstrating that higher hardness enhances durability. Wang et al.^3^ investigated the impact of drilling variables on both conventional and ultrasonic-assisted drilling, revealing greater damage caused by the latter. Mokas et al.^4^ focused on the influence of cutting parameters, highlighting feed rate as the most critical factor affecting wear in high-speed steel (HSS) drills. Singh et al.^5^ provided insights into stress and deformation, showing that maximum stresses occur at the margin, cutting edge, or lip of the drill bit.

These studies collectively emphasize the importance of numerical methods, particularly in analyzing structural mechanics. Among these methods, the finite element method (FEM)^6–8^ is widely recognized for its ability to model complex engineering problems. Despite its strengths, FEM often struggles with challenges such as large deformations, nonlinear materials, and nonuniform boundary conditions. Tetrahedral FEM models, in particular, can exhibit slower convergence and reduced accuracy in stress analysis^9^. Additionally, FEM’s inherent stiffness may result in volume locking, which compromises precision. To address these limitations, researchers have proposed advanced methods. For instance, Rong et al.^10^ introduced an innovative approach to mitigate volume locking, while Doll et al.^11^ extended selective reduced integration (SRI) to elastic-plastic problems, successfully eliminating locking effects. Atluri et al.^12^ developed meshless methods to enhance precision in solving displacement and strain variables, while Wells et al.^13^ and Nicolazzi et al.^14^ introduced alternative techniques to address similar challenges.

Further advancements were made by Liu et al.^15^ with the introduction of the weakened weak form (W²) and the smooth finite element method (S-FEM)^16,17^, which employ gradient smoothing techniques to improve computational efficiency and accuracy. Variants of S-FEM, including NS-FEM^18–20^, ES-FEM^21–23^ and FS-FEM^24,25^, have been developed to tackle specific numerical issues. For example, NS-FEM provides an upper-bound solution with super-convergent stress results but may exhibit time-response instability. Conversely, ES-FEM offers lower-bound solutions with high accuracy^26^ and has been successfully combined with other smoothing methods to enhance performance. Zeng et al.^27^ introduced *β*FEM, which demonstrated exceptional stability and precision.

Building on these developments, this study proposes the *λ*S-FEM method to address the mechanical analysis of 3D twist drills. By integrating NS-FEM and ES-FEM through strain-smoothing techniques, the *λ*S-FEM method is designed to overcome the limitations of traditional FEM while enhancing accuracy. Since both the NS-FEM and ES-FEM methods for tetrahedral elements are spatially stable, the proposed S-FEM method will also be spatially stable^28^, ensuring convergence. This paper is organized as follows: Section "Theory of *λ*S-FEM" outlines the static modeling processes of NS-FEM, ES-FEM, and the theoretical foundation of *λ*S-FEM. Section "Numerical validation of *λ*S-FEM analysis method" validates the method through numerical case studies involving flange and shell extractor components. Section "Application of *λ*S-FEM in mechanical analysis of twist drills" investigates the mechanical properties of twist drills using *λ*S-FEM, while Section "Conclusion" concludes with key findings.

## Theory of *λ*S-FEM

### Theory of ES-FEM and NS-FEM

#### Creation of smooth domains

**Fig. 1:**
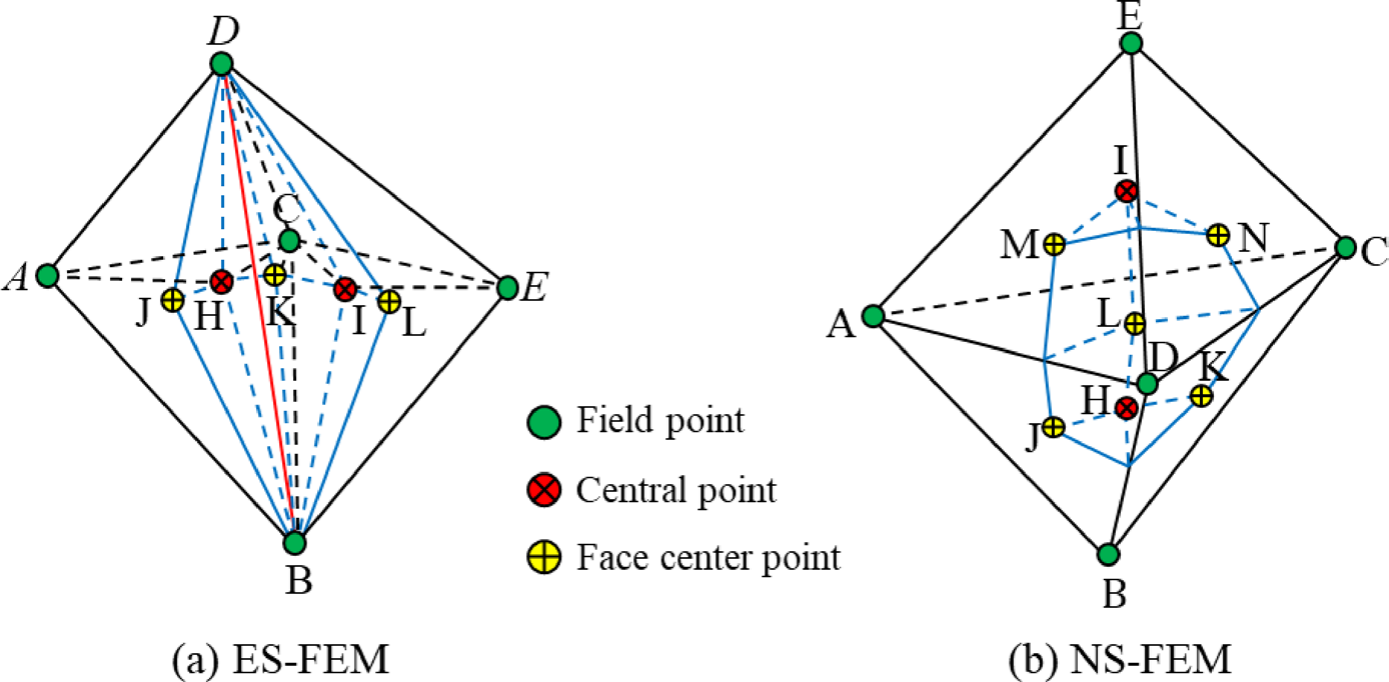
The smooth domains of ES-FEM and NS-FEM.

The element grid for S-FEM is generated using the same approach as that of conventional FEM. It consists of a grid containing ${N_n}$ nodes, ${N_{eg}}$ edges, and ${N_e}$ elements. Based on this element grid, the problem domain $\Omega$ is discretized into ${N_s}$ gapless smooth domains $\Omega _{k}^{s}$, which satisfy conditions $\Omega = \cup _{{k=1}}^{{Ns}}\Omega _{k}^{s}$ and $\Omega _{i}^{s} \cap \Omega _{j}^{s}=\emptyset ,i \ne j$. Subsequently, the smoothing domain was further delineated using a grid of T4 elements. The edge-based smoothing domain is formed by connecting the vertices (B, D) of the edges to the centers of the shapes (H, I) of the neighboring elements, as well as to the centers of the faces (H, K, L) associated with the edges, as illustrated in Fig. 1a. In contrast, the node-based smooth domain is established by linking the midpoints of each edge connected to the node (D), the centers of the shapes (H, I) of the neighboring elements, and the centers of the faces (J, K, L, M, N) related to the node, as illustrated in Fig. 1b.

#### The formulation of smooth strain matrices

The smooth strain field in S-FEM is obtained by calculating the boundary integral of the smooth domain using the following expression:

1 $${{\bar {\varvec{\epsilon }}}_k}=\frac{1}{{V_{k}^{s}}}\int\limits_{{\Omega _{k}^{s}}}^{{}} {{\mathbf{L} _{d}}\bar {\mathbf{u}}\left( \mathbf{x} \right)} d\Omega =\frac{1}{{V_{k}^{s}}}\sum\limits_{{q=1}}^{{{n_s}}} {\int\limits_{{\Omega _{{k,q}}^{s}}}^{{}} {{\mathbf{L} _d}\bar {\mathbf{u}}\left( \mathbf{x} \right)} d\Omega } =\frac{1}{{V_{k}^{s}}}\sum\limits_{{q=1}}^{{{n_s}}} {\int\limits_{{\Gamma _{{k,q}}^{s}}}^{{}} {{\mathbf{L} _n}\bar {\mathbf{u}}\left( \mathbf{x} \right)} d\Gamma }$$

where $V_{k}^{s}$ denotes the volume of the smooth domain $\Omega _{k}^{s}$, $\Gamma _{{k,q}}^{s}$ denotes the boundary of the smooth domain $\Omega _{{k,q}}^{s}$, ${\mathbf{L} _n}\left( \mathbf{x} \right)$ is the matrix of the outer normal components on the boundary $\Gamma _{{k,q}}^{s}$, $\bar {\mathbf{u}}\left( \mathbf{x} \right)$ denotes the displacement field function, ${\mathbf{L} _n}\left( \mathbf{x} \right)$ and $\bar {\mathbf{u}}\left( \mathbf{x} \right)$ are in the form:

2 $${\mathbf{L} _n}\left( \mathbf{x} \right)=\left[ {\begin{array}{*{20}{c}} {{n_x}}&0&0 \\ 0&{{n_y}}&0 \\ 0&0&{{n_z}} \\ 0&{{n_z}}&{{n_y}} \\ {{n_z}}&0&{{n_x}} \\ {{n_y}}&{{n_x}}&0 \end{array}} \right]$$

3 $$\bar{\mathbf{u}}\left( \mathbf{x} \right)=\sum\limits_{{I=1}}^{{{N_n}}} {{\mathbf{N} _I}\left( \mathbf{x} \right)} {\bar {\mathbf{d}}_I}=\left\{ {{\mathbf{N} _1}\left( \mathbf{x} \right){\text{ }}{\mathbf{N} _2}\left( \mathbf{x} \right){\text{ }} \cdot \cdot \cdot {\text{ }}{\mathbf{N} _{{N_n}}}\left( \mathbf{x} \right)} \right\}\left\{ {\begin{array}{*{20}{c}} {{{\bar {\mathbf{d}}}_1}} \\ {{{\bar {\mathbf{d}}}_2}} \\ \vdots \\ {{{\bar {\mathbf{d}}}_{{N_n}}}} \end{array}} \right\}=\mathbf{N} \left( \mathbf{x} \right)\bar {\mathbf{d}}$$

${\mathbf{N} _I}\left( \mathbf{x} \right)$ represents ${N_n}$ linearly independent node shape functions and ${\bar {\mathbf{d}}_I}$ represents the node displacement vector of node *I*.

The discrete control equation is given as follows:

4 $${\bar {\mathbf{K}}^{{\text{ES-FEM/NS-FEM}}}}\bar {\mathbf{d}}=\tilde {\mathbf{f}}$$

where ${\bar {\mathbf{K}}^{{\text{ES-FEM/NS-FEM}}}}$ represents the smooth stiffness matrix, which is expressed as:

5 $$\bar{\mathbf{K}}_{{IJ,i}}^{{{\text{ES - FEM}}}} = \int\limits_{\Omega } {\bar{\mathbf{B}}_{I}^{T} \mathbf{D}\bar{\mathbf{B}}_{J} } d\Omega = \sum\limits_{{i = 1}}^{{N_{{eg}} }} {\int\limits_{{\Omega _{i}^{s} }} {\bar{\mathbf{B}}_{I}^{T} \mathbf{D}\bar{\mathbf{B}}_{J} } d\Omega } = \sum\limits_{{i = 1}}^{{N_{{eg}} }} {\bar{\mathbf{B}}_{I}^{T} \mathbf{D}\bar{\mathbf{B}}_{J} } V_{i}^{s}$$

6 $$\bar{\mathbf{K}}_{{IJ,k}}^{{{\text{NS - FEM}}}} = \int\limits_{\Omega } {\bar{\mathbf{B}}_{I}^{T} \mathbf{D}\bar{\mathbf{B}}_{J} } d\Omega = \sum\limits_{{k = 1}}^{{N_{n} }} {\int\limits_{{\Omega _{k}^{s} }} {\bar{\mathbf{B}}_{I}^{T} \mathbf{D}\bar{\mathbf{B}}_{J} } d\Omega } = \sum\limits_{{k = 1}}^{{N_{n} }} {\bar{B}_{I}^{T} \mathbf{D}\bar{\mathbf{B}}_{J} } V_{k}^{s}$$

**D** represents the matrix of material constants, determined by the Young’s modulus *E* and Poisson’s ratio *ν* of the material:

7 $$\mathbf{D} =\left[ {\begin{array}{*{20}{c}} {{c_{11}}}&{{c_{12}}}&{{c_{12}}}&0&0&0 \\ {}&{{c_{11}}}&{{c_{12}}}&0&0&0 \\ {}&{}&{{c_{11}}}&0&0&0 \\ {}&{}&{}&{\frac{{{c_{11}} - {c_{12}}}}{2}}&0&0 \\ {}&{sy.}&{}&{}&{\frac{{{c_{11}} - {c_{12}}}}{2}}&0 \\ {}&{}&{}&{}&{}&{\frac{{{c_{11}} - {c_{12}}}}{2}} \end{array}} \right]$$

$${c_{11}}=\frac{{E\left( {1 - v} \right)}}{{\left( {1 - 2v} \right)\left( {1+v} \right)}},{c_{12}}=\frac{{Ev}}{{\left( {1 - 2v} \right)\left( {1+v} \right)}}.$$

The global smooth strain matrix $\bar {\mathbf{B}}$ is calculated by the following equation:

8 $${\bar {\mathbf{B}}_I}=\frac{1}{{V_{k}^{s}}}\int\limits_{{\Gamma _{k}^{s}}} {{\mathbf{L} _n}\left( \mathbf{x} \right)} {\mathbf{N} _I}\left( \mathbf{x} \right)d\Gamma =\left[ {\begin{array}{*{20}{c}} {{{\bar {b}}_{Ix}}}&0&0 \\ 0&{{{\bar {b}}_{Iy}}}&0 \\ 0&0&{{{\bar {b}}_{Iz}}} \\ 0&{{{\bar {b}}_{Iz}}}&{{{\bar {b}}_{Iy}}} \\ {{{\bar {b}}_{Iz}}}&0&{{{\bar {b}}_{Ix}}} \\ {{{\bar {b}}_{Iy}}}&{{{\bar {b}}_{Ix}}}&0 \end{array}} \right]$$

9 $${\bar {b}_{Ih}}=\frac{1}{{V_{k}^{s}}}\int\limits_{{\Gamma _{k}^{s}}} {{n_h}} \left( \mathbf{x} \right){N_I}\left( \mathbf{x} \right)d\Gamma \xrightarrow{{{\text{Simplification of Gaussian Integrals}}}}{\bar {b}_{Ih}}=\frac{1}{{V_{k}^{s}}}\sum\limits_{{p=1}}^{{n_{\Gamma }^{S}}} {{n_{h,p}}} {N_I}\left( {x_{p}^{G}} \right){s_p}{\text{ }}\left( {h=x,y,z} \right)$$

$n_{\Gamma }^{S}$ represents the total number of boundary surfaces $\Gamma _{{k,p}}^{s} \in \Gamma _{k}^{s}$, $x_{p}^{G}$ represents the face center (Gauss point) of the boundary surface $\Gamma _{{k,p}}^{s}$, ${s_p}$ represents the area of the boundary surfaces, ${n_{h,p}}$ is the vector of outer normals of the boundary surfaces, and $V_{k}^{s}$ represents the volume of the smooth domain defined by the supporting edges or supporting nodes. The smooth strain matrix in the T4 element discrete 3D problem can be assembled as follows:

10 $${\bar {\mathbf{B}}_I}\left( {{\mathbf{x} _k}} \right)=\frac{1}{{V_{k}^{s}}}\sum\limits_{{j=1}}^{{n_{k}^{e}}} {\frac{1}{4}V_{j}^{e}} \tilde {\mathbf{B}}_{j}^{e}$$

$\tilde {\mathbf{B}}_{j}^{e}=\sum\limits_{{I \in S_{j}^{e}}} {{{\tilde {\mathbf{B}}}_I}}$ denotes the compatible strain matrix of the *j*th T4 element associated with edge or node *k*, $n_{k}^{e}$ denotes the number of T4 elements surrounding edge or node *k*, and $V_{j}^{e}$ denotes the volume of the *j*th T4 element surrounding edge or node *k*. $V_{k}^{s}$ denotes the volume of the *k*th smooth domain, which is expressed as:

11 $$V_{k}^{s}=\int\limits_{{\Omega _{k}^{s}}} {d\Omega =\frac{1}{4}} \sum\limits_{{j=1}}^{{n_{k}^{e}}} {V_{j}^{e}}$$

### Theory of *λ*S-FEM

The *λ*S-FEM fully utilizes the characteristics of NS-FEM in generating the upper bound solution and ES-FEM in generating the lower bound solution by optimizing the adjustable scale factor $\lambda$ to obtain a solution that approximates the exact solution. When the parameter $\lambda =0.0$, *λ*S-FEM becomes NS-FEM, and the strain energy $\hat {E}\left( {\lambda =0} \right)$ is the upper bound solution of the exact solution, and when the parameter $\lambda =1.0$, *λ*S-FEM becomes ES-FEM, and the strain energy $\hat {E}\left( {\lambda =1} \right)$ is the lower bound estimation of the exact strain energy. When $\lambda$ is varied between 0 and 1, the solution of *λ*S-FEM-T4 is a continuous function that interpolates between the NS-FEM and ES-FEM solutions. To validate *λ*S-FEM (T4, $\lambda$) and obtain the optimal range of values for $\lambda$, the strain energy $E\left( \lambda \right)$ is defined as:

12 $$E\left( \lambda \right)=\frac{1}{2}{\mathbf{d} ^T}{\bar {\mathbf{K}}^{\lambda {\text{S-FEM}}}}\mathbf{d}$$

**Fig. 2:**
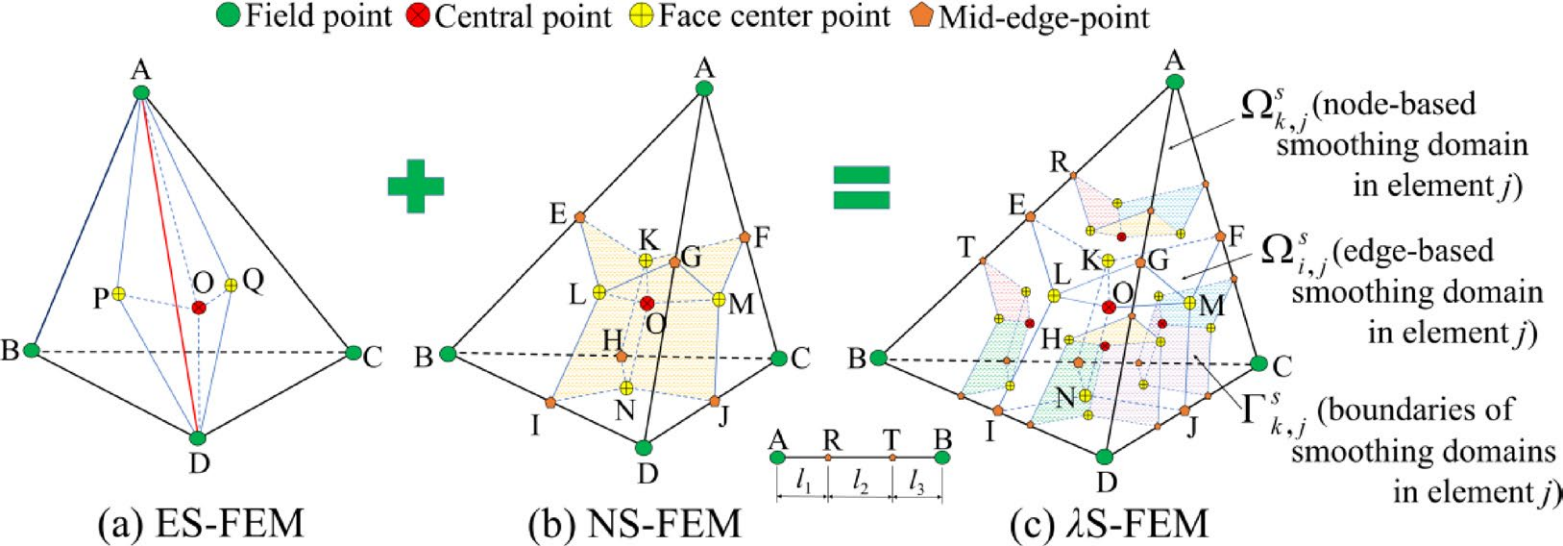
*λ*S-FEM-T4 element composed of tetrahedral elements of ES-FEM and NS-FEM.

This paper uses the *λ*S-FEM-T4 model with tetrahedral elements to address three-dimensional problems. The background elements are categorized into two types of smoothing domains, and the volume part of the smooth domains will be adjusted by parameters $\lambda$. The volume region $\Omega _{i}^{e}$ of the tetrahedral element in *λ*S-FEM-T4 is divided into five subdomains using the adjustable parameter $\lambda$. Each of the four nodes has a subdomain with volume $\left( {1 - {\lambda ^3}} \right)V_{i}^{e}/4$, and the volume of the remaining “Y”-shaped region within the tetrahedral element is denoted as ${\lambda ^3}V_{i}^{e}$, as shown in Fig. 2. Four isovolumetric subdomains were computed using the NS-FEM-T4 model, while the remaining intermediate subdomains were computed using the ES-FEM. In Fig. 2c, assume that the edge lengths of the tetrahedral elements are denoted by L. The proportionality factor $\lambda$ is utilized to adjust the partitioning of points R and T along the edges of AB., and the lengths of the AB edges have the following relationship: ${l_1}={l_{\text{3}}}={1 \mathord{\left/ {\vphantom {1 2}} \right. \kern-0pt} 2}\left( {1 - \lambda } \right)L$ and ${l_2}=\lambda L$.

The smoothed domain volume ${V^\lambda }$ of the *λ*S-FEM consists of node-based smoothed domain volume ${V^N}$ and edge-based smoothed domain volume ${V^E}$:

13 $${V^\lambda }={V^N}+{V^E}$$

14 $${V^N}=\left( {1 - {\lambda ^3}} \right){V^\lambda }{\text{ and }}{V^E}={\lambda ^3}{V^\lambda },\lambda \in \left[ {0,1} \right]$$

Since the volume of the smoothed domain is adjusted by the parameter$$\lambda$$, consequently, the global stiffness matrix $$\overset{\lower0.5em\hbox{$\smash{\scriptscriptstyle\frown}$}}{\mathbf{K}} _{{IJ}}^{{\lambda {\text{S-FEM-T4}}}}$$ of the *λ*S-FEM can be formed by combining $$\bar {\mathbf{K}}_{{IJ,k}}^{{{\text{NS-FEM-T4}}}}$$ and$$\bar {\mathbf{K}}_{{IJ,i}}^{{{\text{ES-FEM-T4}}}}$$:

15 $$\overset{\lower0.5em\hbox{$\smash{\scriptscriptstyle\frown}$}}{\mathbf{K}} _{{IJ}}^{{\lambda {\text{S-FEM-T4}}}}=\sum\limits_{{{\text{k}}=1}}^{{{N_n}}} {\bar {\mathbf{K}}_{{IJ,k}}^{{{\text{NS-FEM-T4}}}}+} \sum\limits_{{i=1}}^{{{N_{{\text{eg}}}}}} {\bar {\mathbf{K}}_{{IJ,i}}^{{{\text{ES-FEM-T4}}}}}$$

For node-based smooth domains, the smooth stiffness matrix $\bar {\mathbf{K}}_{{IJ,k}}^{{{\text{NS-FEM-T4}}}}$ is computed as:

16 $$\bar {\mathbf{K}}_{{IJ,k}}^{{{\text{NS-FEM-T4}}}}=\int\limits_{{\Omega _{k}^{s}}} {\bar {\mathbf{B}}_{I}^{T}\mathbf{D}{{\bar {\mathbf{B}}}_J}} {\text{d}}\Omega =\left( {1 - {\lambda ^3}} \right)\bar {\mathbf{B}}_{I}^{T}\mathbf{D}{\bar {\mathbf{B}}_J}V_{k}^{s}$$

where the volume $V_{k}^{s}$ of the *k*th smoothed domain is calculated using Eq. (11), and the smoothed strain matrix ${\bar {\mathbf{B}}_I}$ is defined as:

17 $${\bar {\mathbf{B}}_I}=\frac{1}{{V_{k}^{s}}}\sum\limits_{{j=1}}^{{n_{k}^{e}}} {\frac{1}{4}\left( {1 - {\lambda ^3}} \right)} V_{{\text{j}}}^{{\text{e}}}\tilde {\mathbf{B}}_{{\text{j}}}^{{\text{e}}}$$

For edge-based smooth domains, the smooth stiffness matrix $\bar {\mathbf{K}}_{{IJ,i}}^{{{\text{ES-FEM-T4}}}}$ is computed as:

18 $$\bar {\mathbf{K}}_{{IJ,i}}^{{{\text{ES-FEM-T4}}}}=\int\limits_{{\Omega _{i}^{s}}} {\bar {\mathbf{B}}_{I}^{T}\mathbf{D}{{\bar {\mathbf{B}}}_J}} {\text{d}}\Omega ={\lambda ^3}\bar {\mathbf{B}}_{I}^{T}\mathbf{D}{\bar {\mathbf{B}}_J}V_{i}^{s}$$

The smooth strain matrix ${\bar {\mathbf{B}}_I}$ and the volume $V_{i}^{s}$ of the *i*th smooth domain have the following expressions:

19 $${\bar {\mathbf{B}}_I}=\frac{1}{{V_{i}^{s}}}\sum\limits_{{l=1}}^{{n_{i}^{e}}} {\frac{1}{4}{\lambda ^3}} V_{l}^{e}\tilde {\mathbf{B}}_{l}^{e}$$

20 $$V_{i}^{s}=\int\limits_{{\Omega _{i}^{s}}} {d\Omega =\frac{1}{4}} \sum\limits_{{l=1}}^{{n_{i}^{e}}} {V_{i}^{e}}$$

### The *λ*S-FEM analysis process

**Fig. 3:**
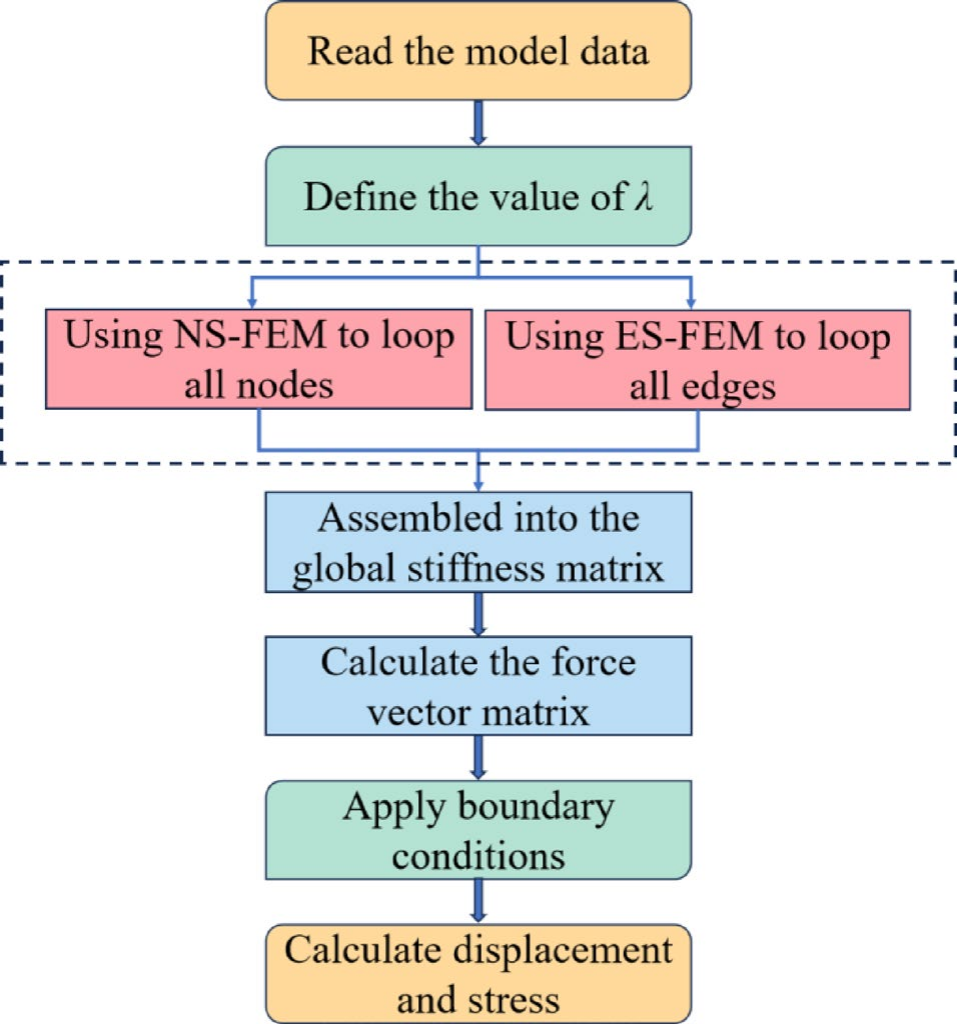
The flow chart of *λ*S-FEM analysis.

The programming procedure to implement the method in MATLAB can be categorized into the following steps: first, import the model and read the data. Then, select the optimal value within the optimal range and define the value $\lambda$. Next, NS-FEM is used to loop over all nodes and ES-FEM is used to loop over all edges. In this step, smooth stiffness matrices based on nodes and edges are computed according to Eqs. (16) and (18), and these two matrices are combined to form the overall stiffness matrix $\bar {\mathbf{K}}={\bar {\mathbf{K}}^{{\text{NS-FEM}}}}+{\bar {\mathbf{K}}^{{\text{ES-FEM}}}}$. Finally, the displacement results are obtained by solving $\bar {\mathbf{K}}\bar {\mathbf{d}}=\tilde {\mathbf{f}}$, which in turn calculates the stresses and preserves the strain energy. The process of force analysis using *λ*S-FEM is shown in Fig. 3.

### Standard patch test

**Fig. 4:**
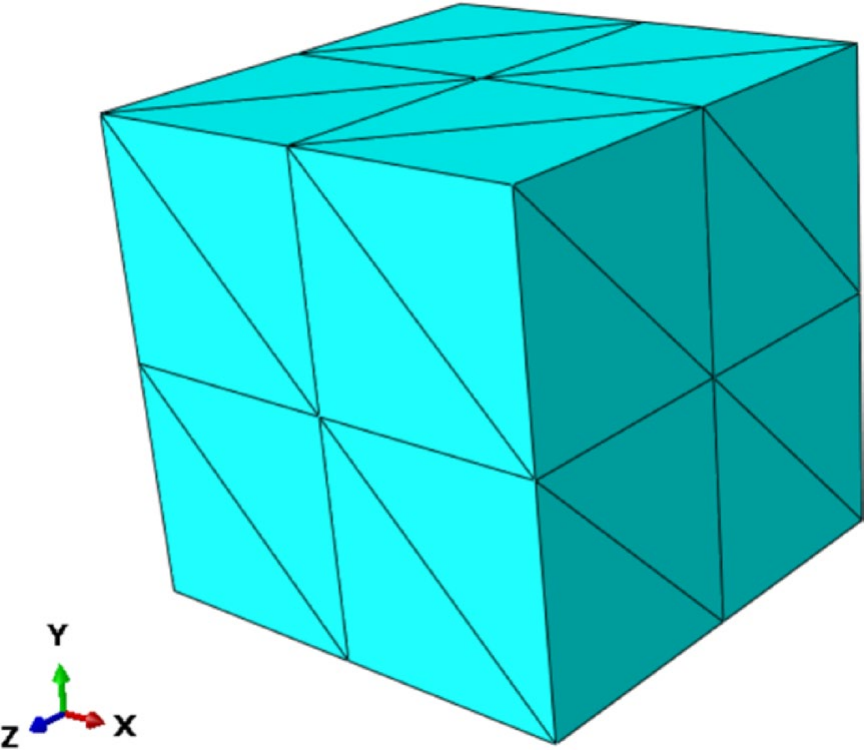
3D cubic patch testing of the *λ*S-FEM using tetrahedral mesh.

In this section, a cube with a side length of 10 mm is used for the patch test, with regional discretization consisting of 96 T4 elements and 35 nodes, as illustrated in Fig. 4. The dimensionless parameters $E=100$ and $v=0.3$ are utilized in the analysis. The following linear displacement field is prescribed on the outer boundary of the cube patch, which includes at least one internal node:

21 $$\begin{gathered} u=2x+y+z \hfill \\ v=x+2y+z \hfill \\ w=x+y+2z \hfill \\ \end{gathered}$$

The analytical experiment satisfies the condition that the displacements of all internal nodes must correspond to the linear function of the applied displacements. Therefore, the numerical results are evaluated using the displacement error metric, which is defined as follows:

22 $${e_d}=\frac{{\sum\nolimits_{{i=1}}^{{{N_{{\text{dof}}}}}} {\left| {{u_i} - {{\bar {u}}_i}} \right|} }}{{\sum\nolimits_{{i=1}}^{{{N_{{\text{dof}}}}}} {\left| {{u_i}} \right|} }} \times 100{\text{\% }}$$

Where ${u_i}$ and ${\bar {u}_i}$ represent the exact and numerical displacements of node *i*, respectively, and ${N_{{\text{dof}}}}$ denotes the total number of degrees of freedom in the system. The displacement error metrics calculated from Eq. (22) are presented in Table 1. From the numerical results in the table, it is evident that the *λ*S-FEM-T4 successfully passes the standard first-order convergence test within the range of mechanical accuracy, regardless of the value of $\lambda \in [0,1]$ chosen.

**Table 1:** Displacement error norm for 3D patch test.

	*λ* = 0 NS-FEM-T4	*λ* = 0.2826(*)	*λ* = 0.5	*λ* = 0.7374(*)	*λ* = 1 ES-FEM-T4
e_*d*_(%)	7.0660e-14	1.1527e-13	1.3027e-13	6.3159e-14	4.6185e-14

(*) Random number.

## Numerical validation of *λ*S-FEM analysis method

Finite element calculation results with fine meshing, using flanges for articulating fastened pipes and shell extractor components in mechanical structures as examples, serve as reference solutions to validate the effectiveness of *λ*S-FEM in addressing solid mechanics problems. In *λ*S-FEM, $\lambda$ takes values within the range $[0,1]$. Select a set of arrays $\lambda \in [0,1]$ such that $\lambda ={[0.0{\text{ }}0.2{\text{ }} \ldots {\text{0.8 1.0}}]^T}$. Iterate through the array to compute the strain energy corresponding to each $\lambda$ value across varying DOF. The results are then plotted as a strain energy curve. By identifying the intersection of the strain energy curve with the reference solution, the optimal range of values for $\lambda$ is determined, from which an optimal value of $\lambda$ is selected.

In numerical computation and algorithm optimization, error reduction serves as a critical measure of the improvement achieved by a newly proposed method. To quantitatively assess the enhancement of *λ*S-FEM, the proportion of error reduction is employed, which is calculated as follows:

23 $$\Delta \varepsilon {\text{=}}\frac{{ErrorA-ErrorB}}{{ErrorA}} \times 100{\text{\% }}$$

### Flange

**Fig. 5:**
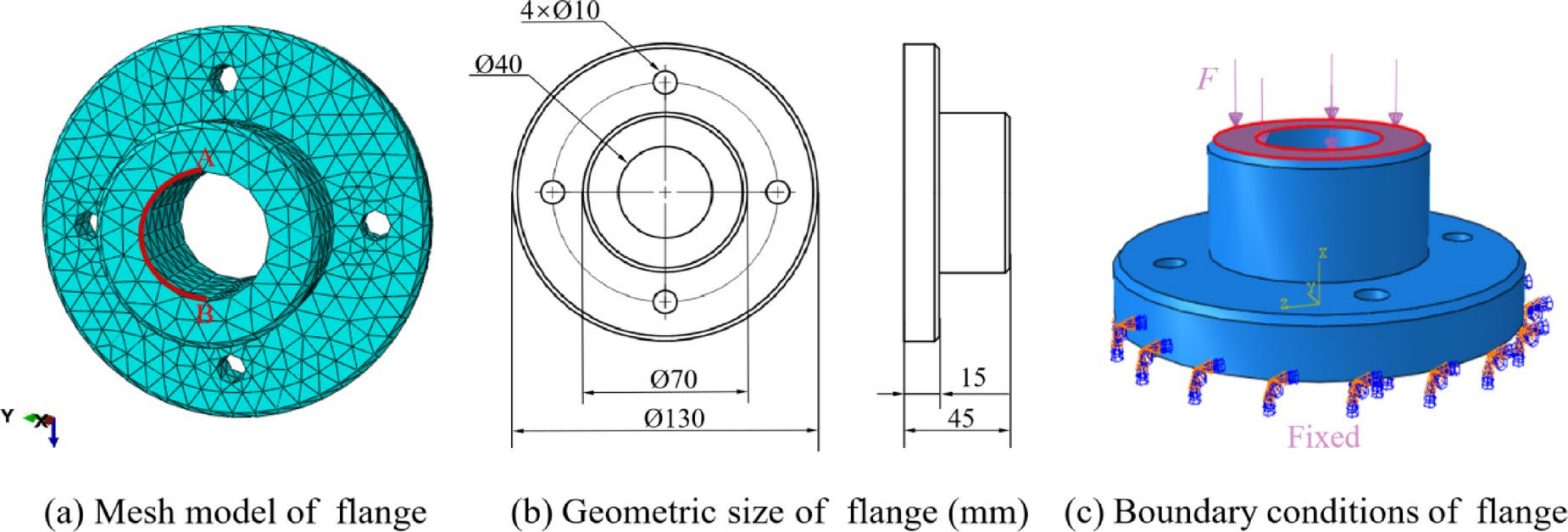
Numerical model of flange.

Flanges are commonly used components in mechanical engineering for connecting pipes and are widely applied in various mechanical systems. In this study, the flange is considered as the research object, with its discrete model, geometry, and boundary conditions depicted in Fig. 5. The material properties of the flange are defined by $E=209,000{\text{MPa}}$, $v=0.269$, and a pressure $F=5 \times {10^4}{\text{N}}$ applied to its top surface. A finite element model comprising 40,321 nodes was used as the reference solution.

**Fig. 6:**
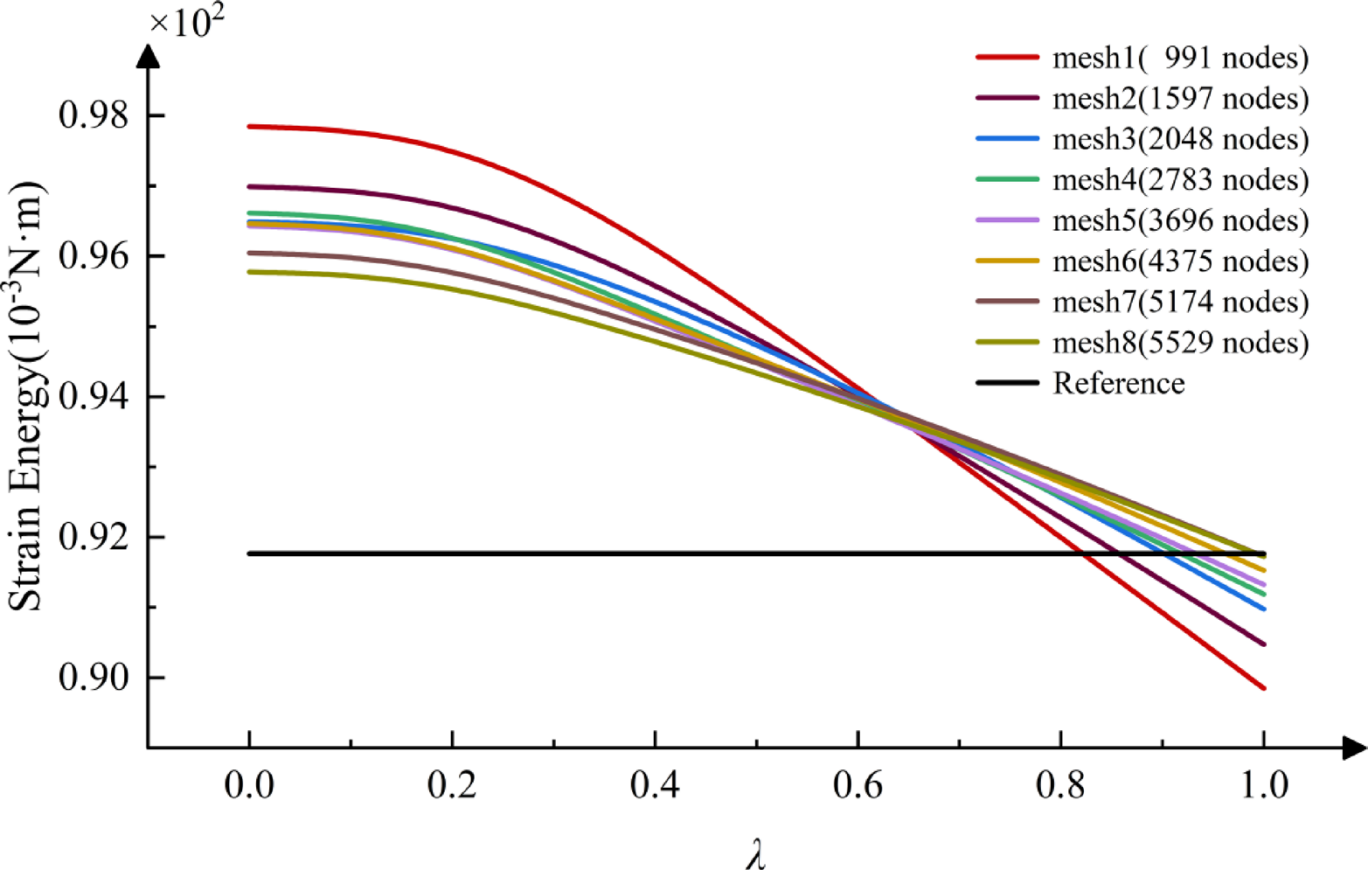
Strain energy curves for flanges with different degrees of freedom.

The variation curves of strain energy with eight different degrees of freedom (mesh information shown in Table 2) are plotted in Fig. 6. The accuracy and convergence of finite element simulations are highly dependent on the quality of the tetrahedral mesh. To assess the mesh quality, this study utilizes HyperMesh software to evaluate the mesh, with the relevant data presented in Table 3. The data indicate that the maximum aspect ratio value for Mesh 1 is 5.64, and 0.16% of the elements exceed an aspect ratio of 5. All other parameters satisfy the required standards, and overall, the mesh quality meets the fundamental requirements for the analysis.

**Table 2:** Different grid types of flanges and related information.

Model	Type	Node	Element	DOF
Model 1	Mesh 1	991	3698	2973
Model 2	Mesh 2	1597	6358	4791
Model 3	Mesh 3	2048	8430	6144
Model 4	Mesh 4	2783	11,915	8349
Model 5	Mesh 5	3696	16,340	11,088
Model 6	Mesh 6	4375	19,551	13,125
Model 7	Mesh 7	5174	23,518	15,522
Model 8	Mesh 8	5529	25,219	16,587
Reference	Mesh 9	40,321	209,311	120,963

**Table 3:** Mesh quality inspection of flanges.

Type	Aspect ratio (Max)	Warpage (Max)	Skew (Max)	Jacobian (Min)
Mesh 1	5.64(6 of 3698)	0	0.89	1
Mesh 2	4.07	0	0.82	1
Mesh 3	3.53	0	0.75	1
Mesh 4	7.02(2 of 11915)	0	0.88	1
Mesh 5	3.2	0	0.77	1
Mesh 6	3.02	0	0.71	1
Mesh 7	3.13	0	0.75	1
Mesh 8	3.25	0	0.71	1
Mesh 9	3.18	0	0.81	1

**Fig. 7:**
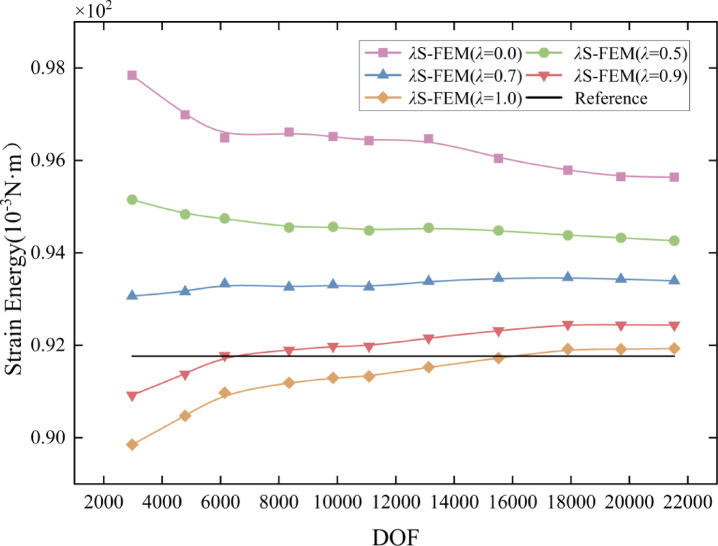
Strain energy convergence curves for flange with different values of $\lambda$.

**Fig. 8:**
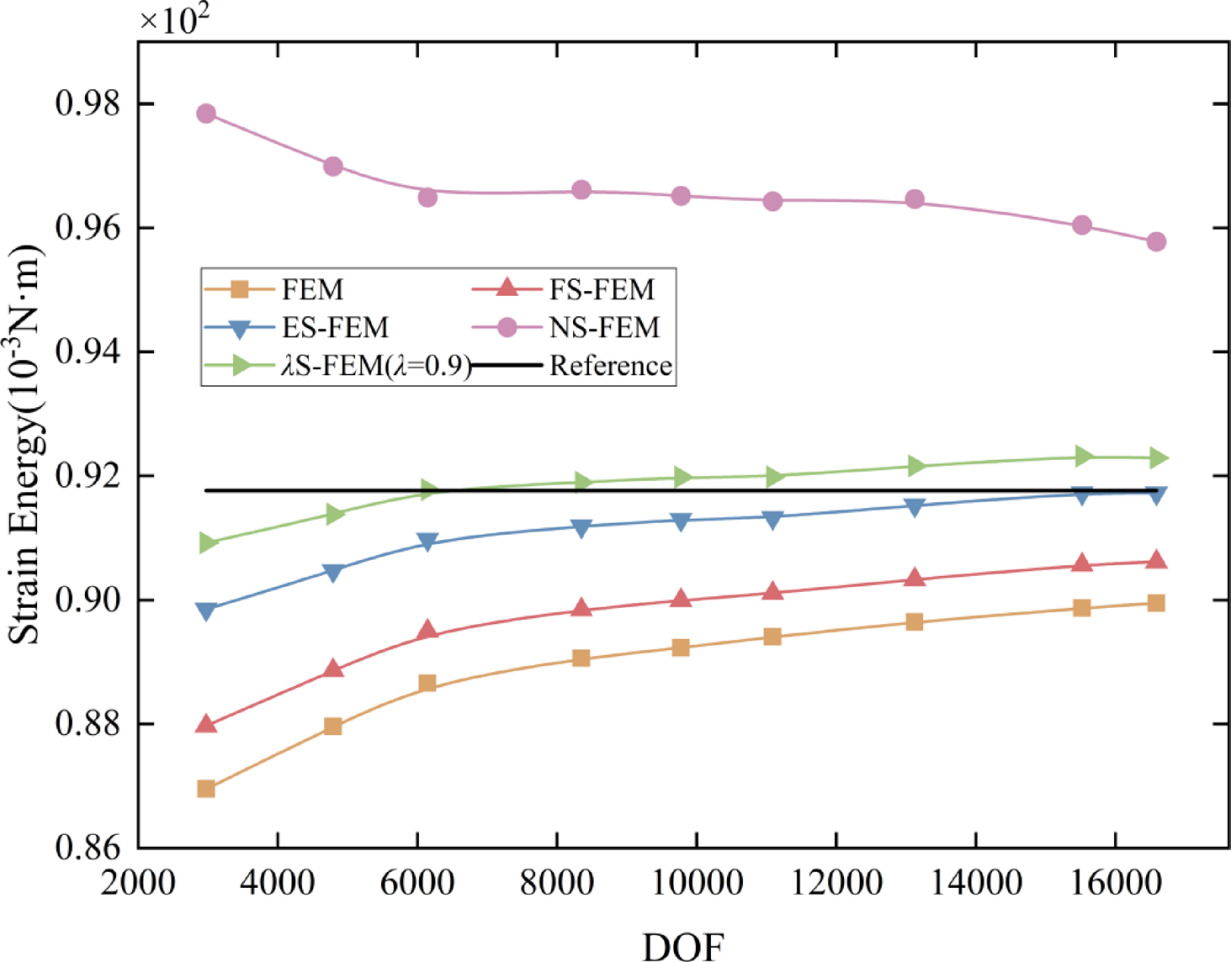
Strain energy convergence curves of flange for five methods.

The optimal range for parameter $\lambda$ is determined to be $[0.8,1]$, based on the intersection of the curve with the reference solution in the Fig. 6. Five distinct sets of $\lambda$ values are selected within the range $[0,1]$, with one set falling within the optimal range. The variation curves of strain energy with respect to the DOF of the *λ*S-FEM for different $\lambda$ values are graphed in Fig. 7. As illustrated in the figure, the *λ*S-FEM at $\lambda =0.9$ yields the most accurate approximation of the solution. Additionally, the strain energy of *λ*S-FEM at $\lambda =0.9$ is compared with that of other S-FEMs and plotted in Fig. 8. The figure illustrates that *λ*S-FEM ($\lambda =0.9$) achieves a 46.5% reduction in strain energy error compared to ES-FEM at low degrees of freedom (fewer than 12,000). Additionally, the strain energy of *λ*S-FEM ($\lambda =0.9$) shows greater alignment the reference solution at a coarse mesh and converges 87% more effectively than ES-FEM. These results demonstrate and validate that the coupled smoothing technique, *λ*S-FEM, proposed in this paper is well-suited for solid mechanics analysis. The optimal parameter $\lambda$ is found to lie within the range $[0.8,1]$, which outperforms other methods for the same degree of freedom.

**Fig. 9:**
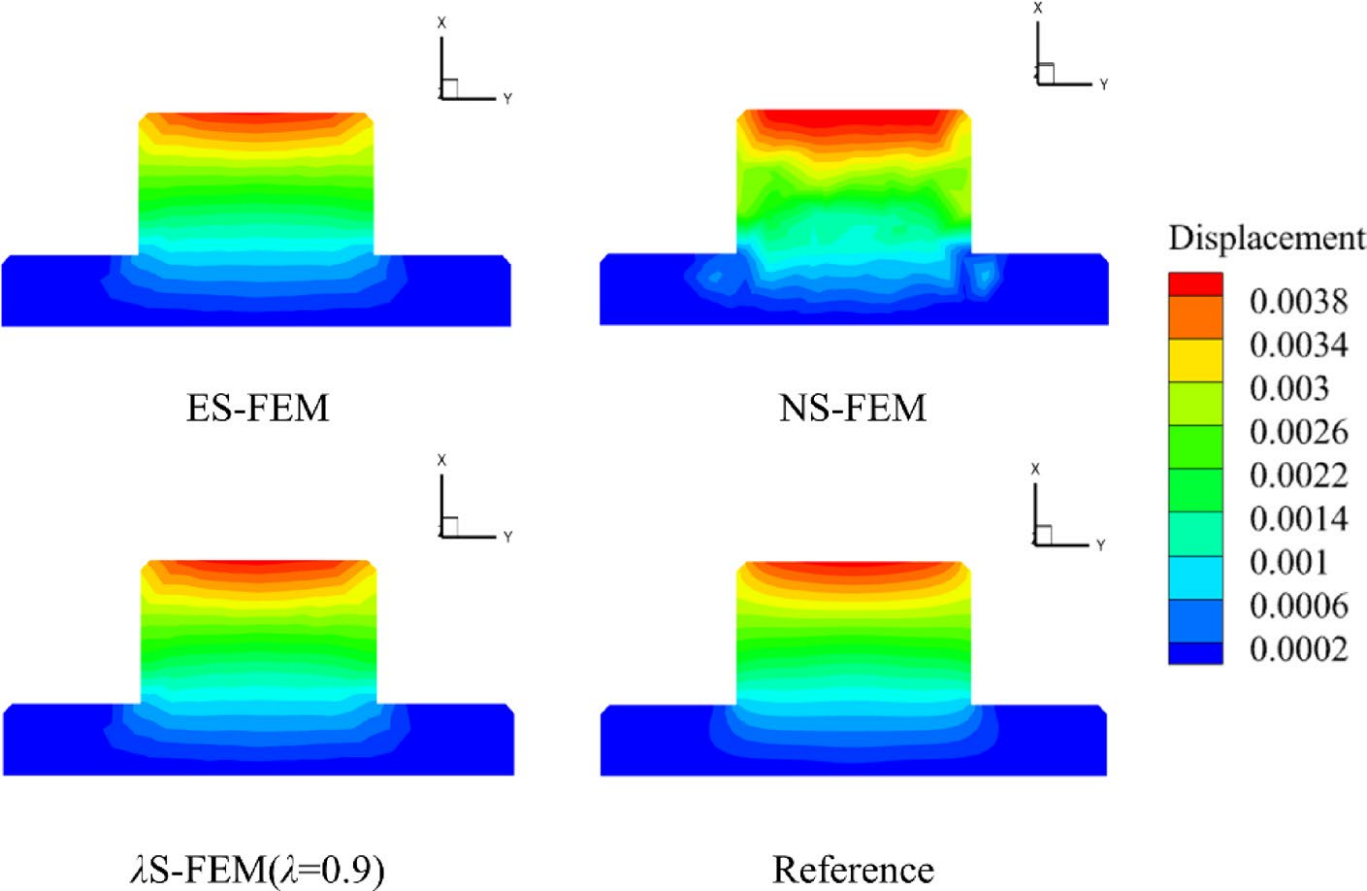
The displacement slice cloud of flange surface (mm).

The numerical results from the five methods are compared on a grid consisting of 6,358 elements, with the surface displacement distribution shown in a cloud diagram, as illustrated in Fig. 9. The reference solution yields a maximum displacement of $3.92 \times {10^{ - 3}}{\text{mm}}$, while the ES-FEM method produces a maximum displacement of $3.88 \times {10^{ - 3}}{\text{mm}}$. In contrast, the NS-FEM method results in a maximum displacement of $4.29 \times {10^{ - 3}}{\text{mm}}$, which deviates significantly from the reference solution. The maximum displacement of *λ*S-FEM ($\lambda =0.9$) is $3.94 \times {10^{ - 3}}{\text{mm}}$, making it the closest to the reference solution.

**Fig. 10:**
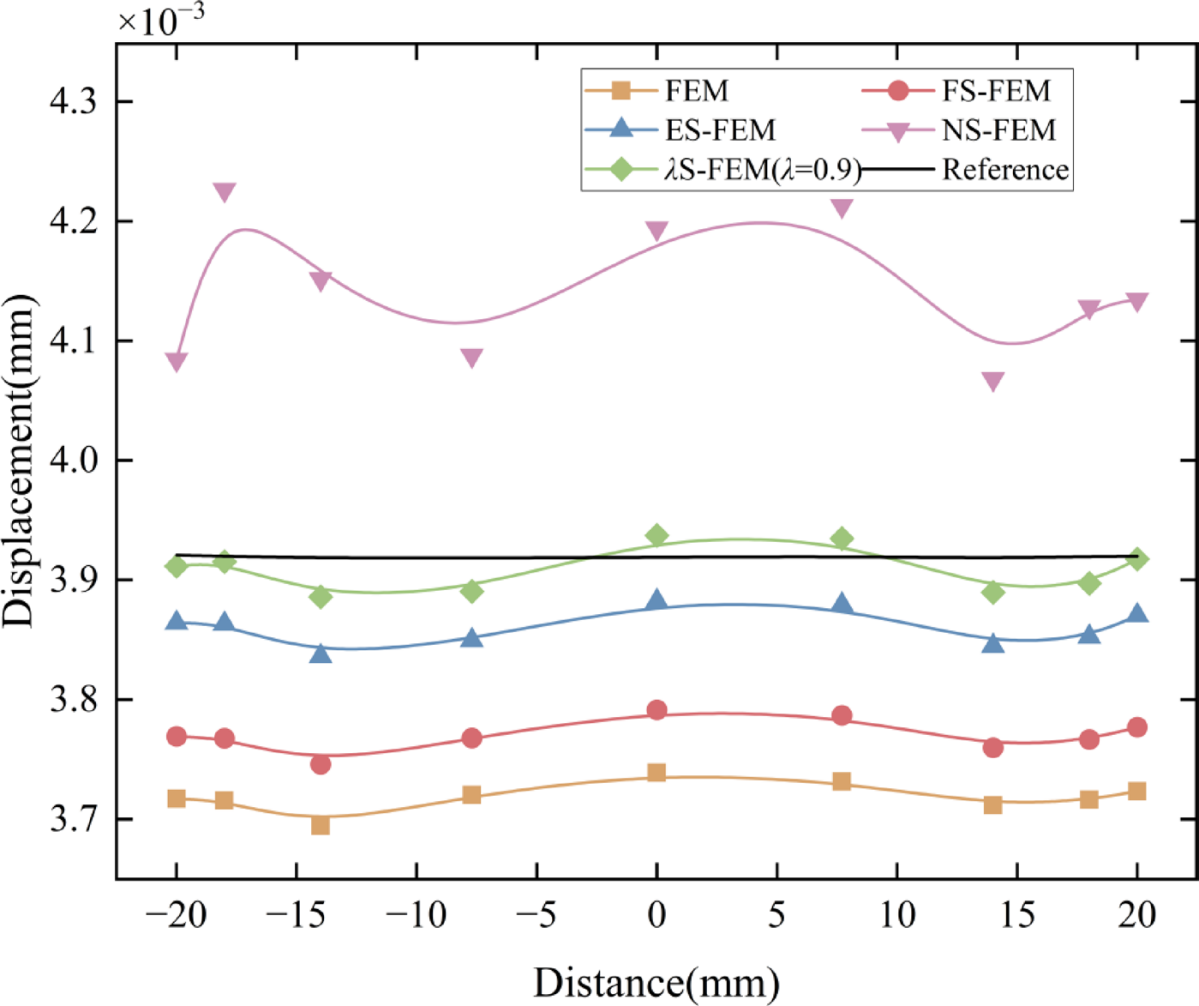
The displacement distribution of flange along the AB arc.

**Fig. 11:**
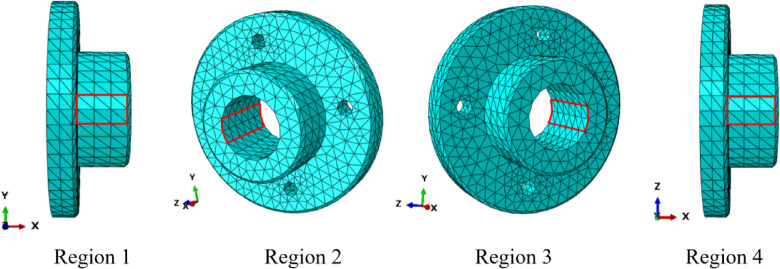
The four sub-regions of the flange model.

**Fig. 12:**
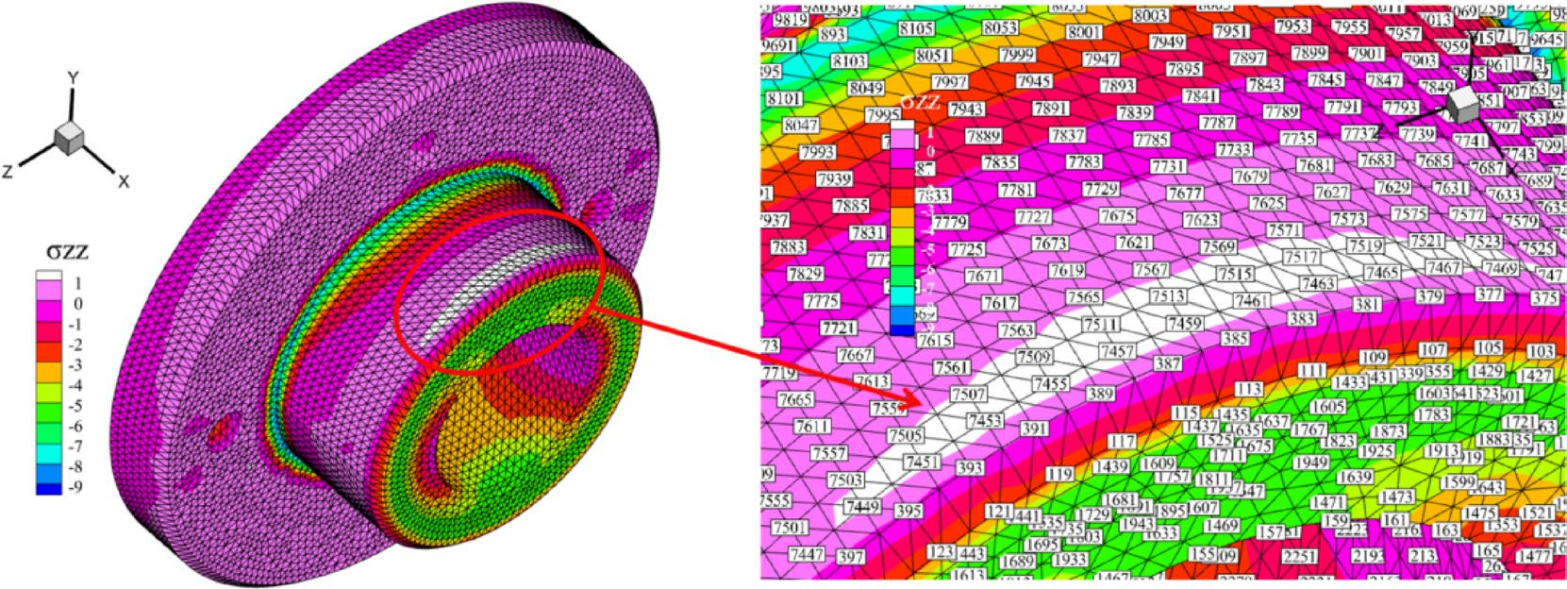
The maximum stress area of the flange.

To visually compare the accuracy of the numerical methods, the nodal displacements along the circular arc AB in Fig. 5a were calculated and are presented in Fig. 10. For model evaluation, the arc AB and the four sub-regions illustrated in Fig. 11 were selected. Displacement errors were computed for each sub-region individually, and the weighted average of these errors (as shown in Table 4) was then calculated to obtain the overall displacement error for the entire region. For the arc AB, the error in *λ*S-FEM ($\lambda =0.9$) is reduced by 69.3% compared to ES-FEM. For the four sub-regions and the overall error, the error reductions in *λ*S-FEM ($\lambda =0.9$) compared to ES-FEM are 13%, 10.97%, 7.77%, 19.55%, and 11.38%, respectively. The stress results for ten randomly selected nodes on the flange were calculated and are summarized in Table 5. Compared to FEM and ES-FEM, *λ*S-FEM ($\lambda =0.9$) reduced the error by 89.3% and 66.1%, respectively, demonstrating a clear advantage over both methods in terms of computational accuracy. The maximum stress region of the flange is depicted in Fig. 12, while the corresponding maximum stress and strain results are presented in Table 6.

**Table 4:** Global displacement error of flanges.

Region	FEM	FS-FEM	ES-FEM	*λ*S-FEM (*λ* = 0.9)	NS-FEM
Region 1	0.0666	0.0523	0.0313	0.0272	0.2053
Region 2	0.0489	0.0405	0.0269	0.0240	0.1126
Region 3	0.0533	0.0444	0.0311	0.0287	0.1176
Region 4	0.0628	0.0457	0.0201	0.0162	0.1373
Global error	0.0561	0.0448	0.0278	0.0246	0.1355

**Table 5:** The stress results of the flange (N/mm^2^).

Point	1	2	3	4	5	6	7	8	9	10
FEM	0.26	0.23	0.24	0.24	0.23	0.22	0.21	0.22	0.23	0.18
FS-FEM	0.25	0.22	0.22	0.22	0.21	0.20	0.19	0.20	0.20	0.18
ES-FEM	0.23	0.20	0.20	0.19	0.19	0.18	0.18	0.18	0.18	0.16
*λ*S-FEM ($\lambda =0.9$)	0.22	0.19	0.19	0.18	0.17	0.17	0.17	0.17	0.16	0.15
NS-FEM	0.36	0.31	1.31	0.23	0.37	0.52	0.59	0.19	0.84	0.18
Reference	0.22	0.19	0.19	0.18	0.17	0.17	0.17	0.16	0.16	0.12

**Table 6:** Maximum stress and strain results of the flange (N/mm^2^).

Method	Maximum stress (σ_zz_)	Maximum strain (ɛ_zz_)
FEM	0.34	3.27E-05
FS-FEM	0.46	3.33E-05
ES-FEM	0.88	3.39E-05
*λ*S-FEM ($\lambda =0.9$)	1.18	3.47E-05
NS-FEM	3.14	4.17E-05
Reference	1.77	3.81E-05

### Shell extractor component

**Fig. 13:**
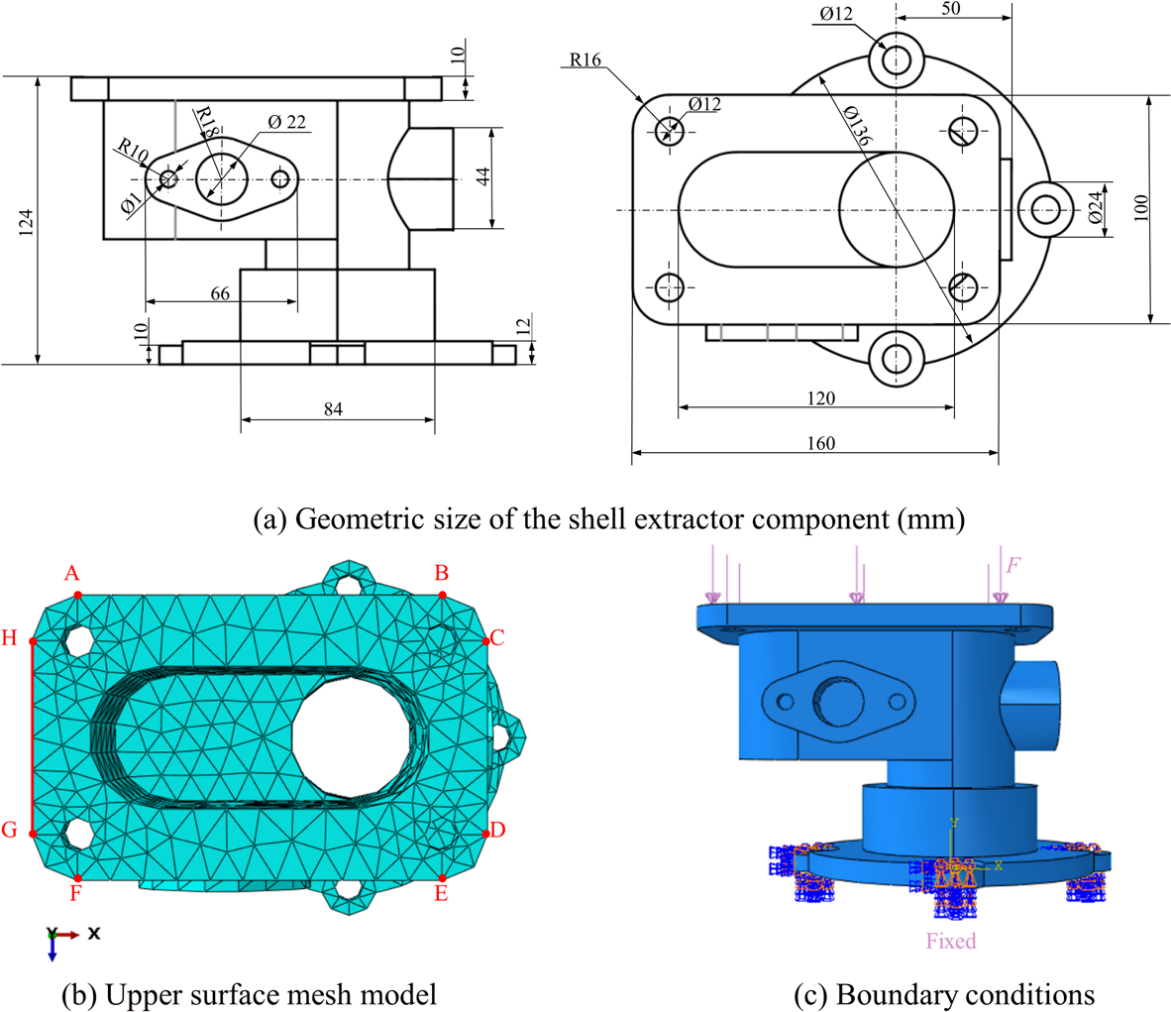
Geometric modeling of the shell extractor component.

Mechanical analysis is conducted on an extracted shell component, with the geometric model, discrete model, and boundary conditions depicted in Fig. 13. A pressure $F=500{\text{N}}$ is applied to the upper surface of the component, while the material properties are characterized by $E=69,000{\text{MPa}}$, $v=0.33$. The solution obtained from the FEM calculation, using a mesh consisting of 167,846 T4 elements, is considered the reference solution. Based on the optimal value range determined from the flange numerical case, a value of $\lambda =0.964$ within this range is selected for the calculation and analysis of the shell extractor component.

**Fig. 14:**
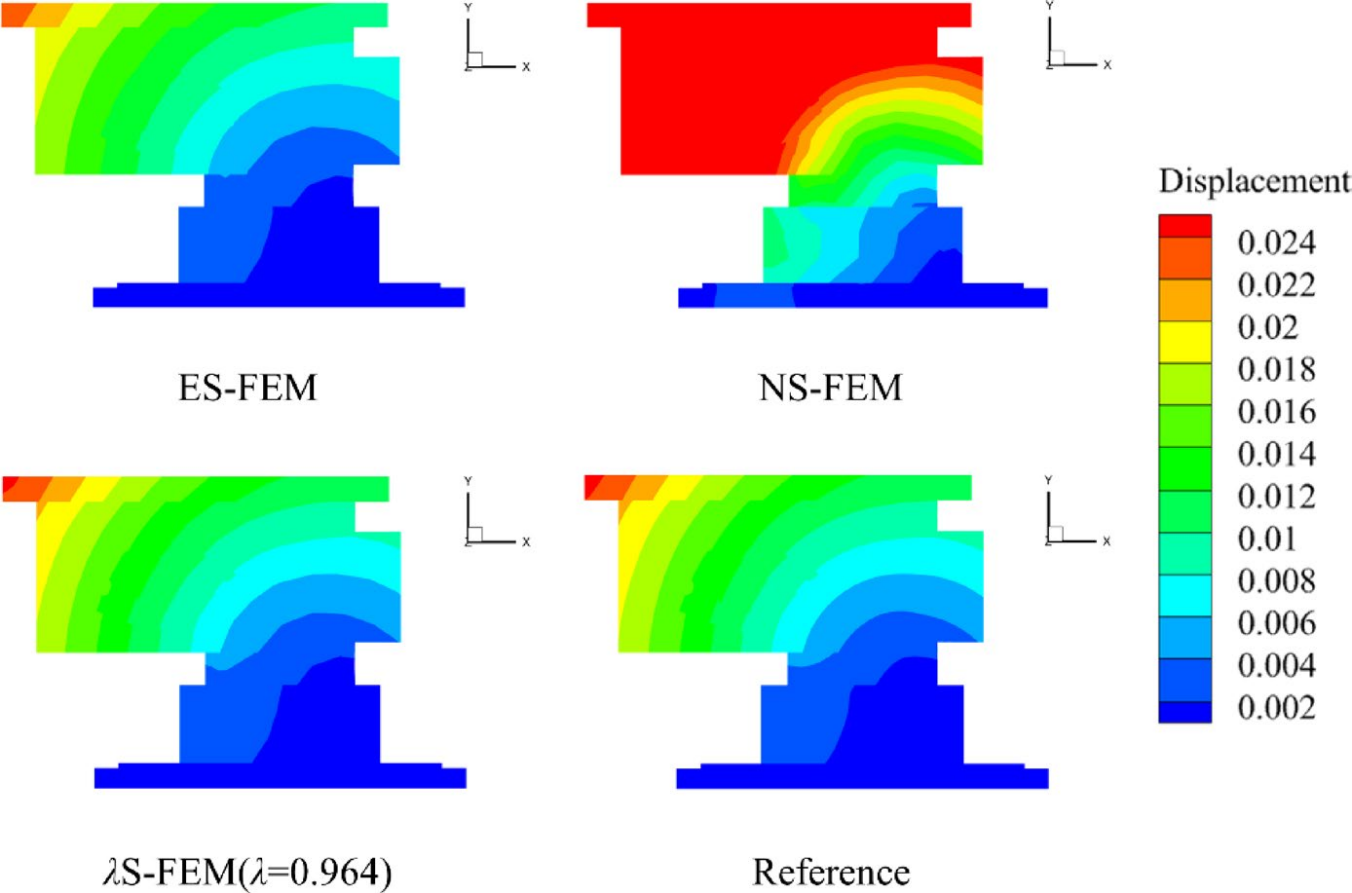
Comparison of surface displacement cloud maps of the shell extractor component (mm).

**Fig. 15:**
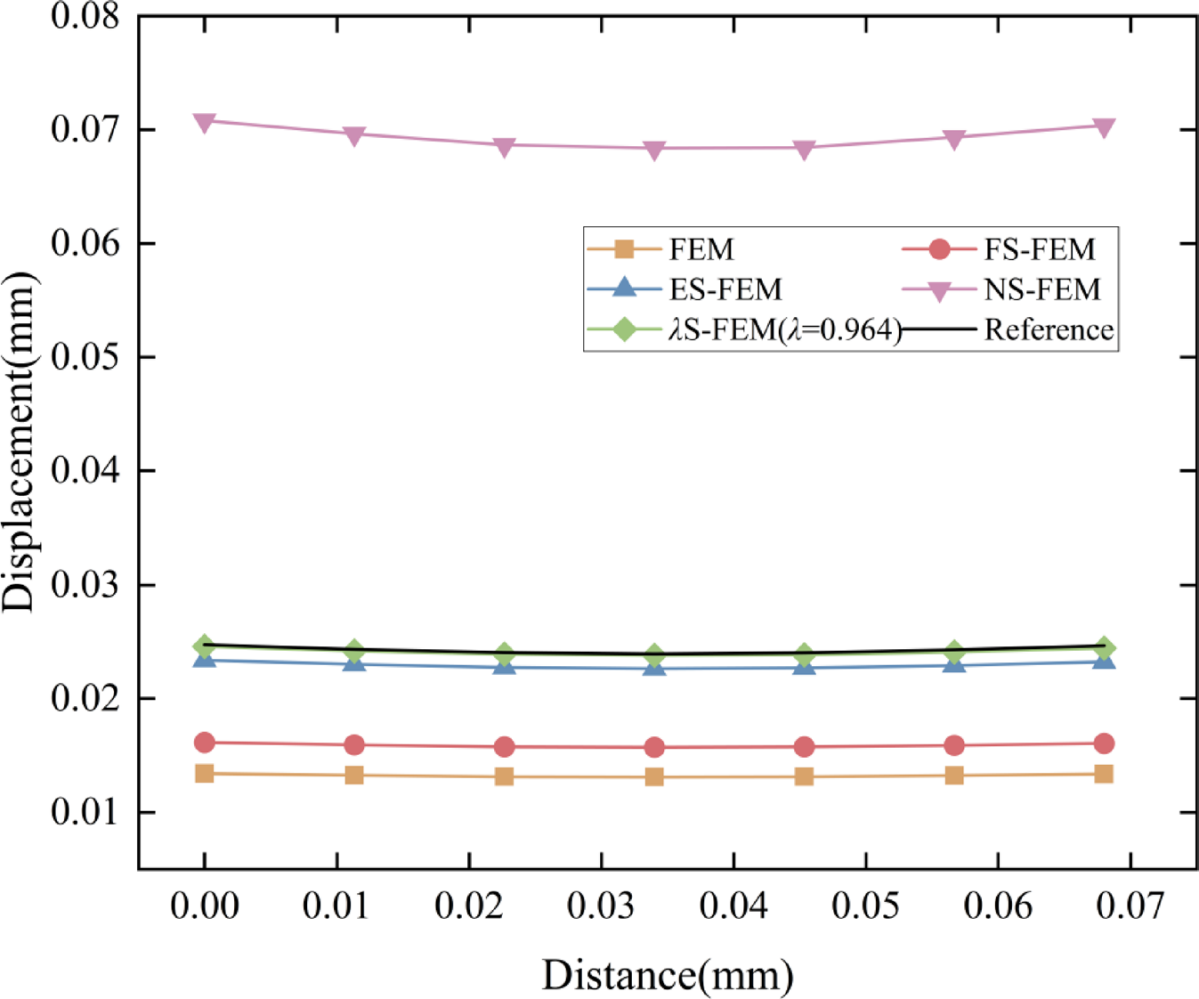
Distribution of node displacements along GH edge.

The numerical results from the five methods were compared on a grid consisting of 4,509 elements, with the surface displacement distribution depicted in Fig. 14. Similarly, to facilitate a more intuitive comparison of the accuracy of five numerical methods, the displacements at the GH edge nodes in Fig. 13b are analyzed. The displacement distribution along the GH edge nodes is presented in Fig. 15. Owing to the “soft” stiffness matrix of the NS-FEM, the results are consistent with previous numerical examples and are greater than the reference values. The results demonstrate that the *λ*S-FEM ($\lambda =0.964$) delivers an optimal approximate solution, eliminating the errors present in ES-FEM and NS-FEM.

**Fig. 16:**
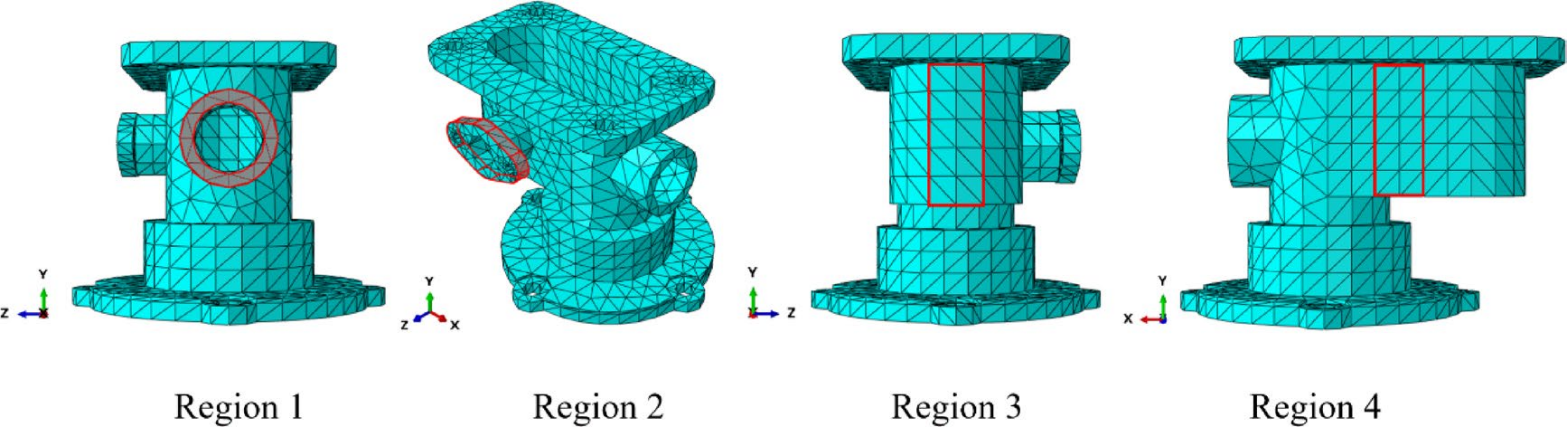
The four sub-regions of the shell extractor component.

**Fig. 17:**
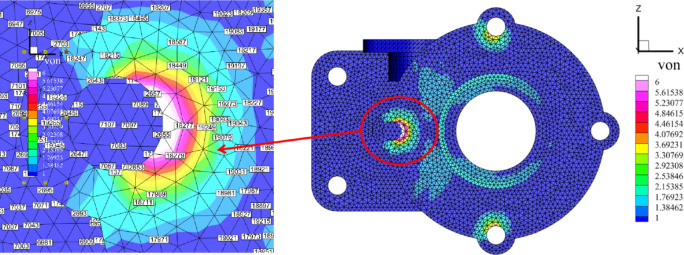
The maximum stress area of the shell extractor component.

The GH edge and the four sub-regions shown in Fig. 16 were selected to compute the displacement errors, with the results for the four sub-regions and the overall region presented in Table 7. The *λ*S-FEM ($\lambda =0.964$) calculations indicated that the displacement error for the GH edge is 12.3% of that for the ES-FEM, while the errors for the four subregions and the overall region are 26.12%, 7.65%, 18.46%, 24.95%, and 18.33% of those for the ES-FEM, respectively. The average displacement error calculated by *λ*S-FEM ($\lambda =0.964$) are 0.37%, 0.88%, 0.2%, 0.59%, 0.77% and 0.55% of NS-FEM, effectively mitigating the bias introduced by the excessively soft stiffness matrix of NS-FEM. Additionally, the stresses at ten random nodes across the entire part are analyzed and presented in Table 8. *λ*S-FEM reduces the stress error by 45.5% and 85.4% compared to ES-FEM and NS-FEM. The region of maximum stress of the shell extractor component is shown in Fig. 17 while the maximum stress and strain results are given in Table 9.

**Table 7:** Global displacement error of the shell extractor component.

Region	FEM	FS-FEM	ES-FEM	*λ*S-FEM (*λ* = 0.964)	NS-FEM
Region 1	0.4861	0.3739	0.0693	0.0181	2.0576
Region 2	0.4648	0.3526	0.0505	0.0039	1.9668
Region 3	0.4556	0.3471	0.0590	0.0109	1.8454
Region 4	0.4774	0.3654	0.0621	0.0155	2.0177
Global error	0.4702	0.3588	0.0587	0.0108	1.9729

**Table 8:** The stress results of the shell extractor component (N/mm^2^).

Point	1	2	3	4	5	6	7	8	9	10
FEM	0.09	0.13	0.20	0.12	0.08	0.09	0.13	0.31	0.18	0.25
FS-FEM	0.10	0.15	0.21	0.13	0.09	0.09	0.14	0.36	0.19	0.30
ES-FEM	0.11	0.17	0.22	0.17	0.11	0.10	0.16	0.44	0.21	0.41
*λ*S-FEM ($\lambda =0.964$)	0.12	0.20	0.26	0.28	0.12	0.12	0.19	0.61	0.26	0.54
NS-FEM	0.27	0.49	0.76	0.98	0.26	0.22	0.60	1.39	0.72	1.66
Reference	0.13	0.29	0.37	0.27	0.15	0.12	0.25	0.67	0.35	0.70

**Table 9:** Maximum stress and strain results of the shell extractor component (N/mm^2^).

Method	Maximum stress (Von)	Maximum strain (ɛ_zz_)
FEM	3.11	9.25E-06
FS-FEM	3.48	1.06E-05
ES-FEM	4.09	1.30E-05
*λ*S-FEM ($\lambda =0.964$)	5.17	2.40E-05
NS-FEM	13.58	7.26E-05
Reference	7.20	2.96E-05

## Application of *λ*S-FEM in mechanical analysis of twist drills

### Physical modeling of twist drill

**Fig. 18:**
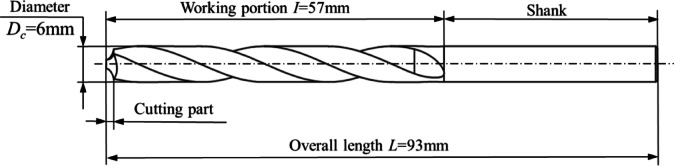
Straight shank twist drill structure.

In this study, a 6 mm straight shank twist drill was used, with its material parameters detailed in Table 10. The selected cutting material was 304 stainless steel, characterized by a Brinell hardness of 187 HB. The drilling process was performed with a feed rate of 120 mm/min and a spindle speed of 800 rpm. The straight shank twist drill comprises a shank and a working part, which includes a cutting section and a guiding section, as illustrated in Fig. 18. The cutting section is primarily responsible for material removal and features two major cutting edges, two minor cutting edges, and a chisel edge. The spiral groove surface acts as the rake face of the drill bit, while the spiral surface near the cutting tip forms the flank face. Additionally, the margin serves as the secondary flank face, and the chisel edge is the intersection of the two main flank faces. During the drilling process, the primary cutting forces include axial and radial forces. The radial force acts perpendicular to the axial direction, transferring the cutting force into energy for material removal. Due to the symmetrical positioning of the drill’s cutting edges, the opposing radial forces cancel each other out, thus preventing axial bending. Axial forces, on the other hand, act along the drill’s axis, enabling the drill bit to penetrate and exit the workpiece. In this study, axial force is applied specifically to the cutting section of the drill. Various S-FEM methods are employed to analyze the mechanical properties of twist drills made from different materials as they engage with the workpiece. These analyses aim to provide insights into the structural behavior and performance of the drills under operational conditions.

**Table 10:** Twist drill material parameters.

Material	*E* (GPa)	*ν*
WC	530	0.31
TiN	600	0.25
M35	207	0.30

The axial force is calculated as:

24 $$F=0.24 \times HB \times {D_c}^{{0.95}} \times {f^{0.61}} \times 9.8$$

where $HB$ represents the Brinell hardness of the material being cut, ${D_c}$ is the sharp edge diameter of the drill, and *f* denotes the feed per rotation.

25 $$f=\frac{{{v_f} \times \pi \times {D_c}}}{{{v_c} \times 1000}}=\frac{{{v_f}}}{n}$$

where ${v_f}$ denotes feed rate, ${v_c}$ denotes cutting speed, and *n* denotes spindle speed.

**Fig. 19:**
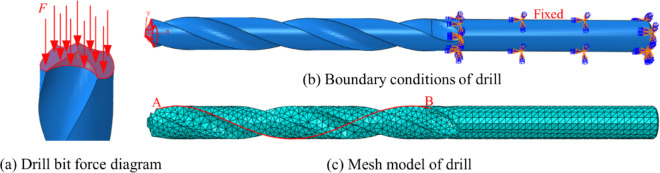
Twist drill boundary conditions and mesh model.

As a result of the analysis, the axial force exerted on the twist drill is calculated to be 518.987 N, which is approximated to 519 N for further calculations. The twist drill model is meshed using standard tetrahedral elements to ensure precise simulation. The boundary conditions and the discrete model for this analysis are depicted in Fig. 19, providing a clear representation of the constraints and setup used in the computational study.

### The process of optimizing the value of *λ*

**Fig. 20:**
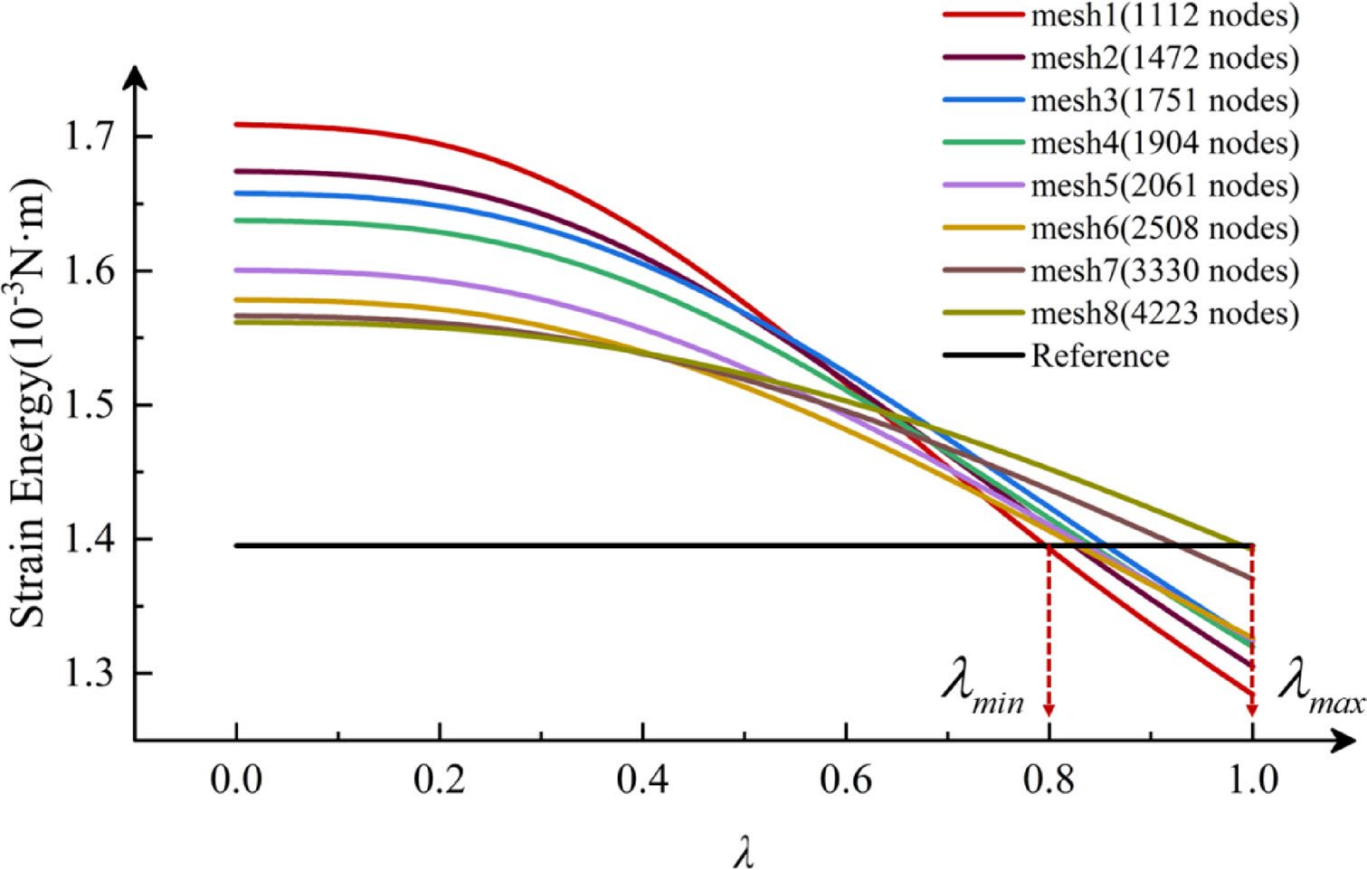
The strain energy curve of 3D twist drill obtained by *λ*.

Numerical simulations are conducted for different drilling conditions of 304 stainless steel using WC, TiN, and M35 twist drills. The refined finite element calculation results are used as reference solutions. Eight background grids were employed to compute the strain energy of $\lambda \in [0,1]$ using five different methods. Details of the eight grids are presented in Table 11, while the mesh quality inspection data in Table 12 demonstrates that the mesh quality of the finite element model is satisfactory and meets the fundamental requirements for finite element analysis. The strain energy, calculated for various DOF as Eq. (12), presented in the form of a strain energy curve, as illustrated in Fig. 20. Furthermore, it is evident from Fig. 20 that the optimal range of values for $\lambda$ is determined to be ${\lambda _{opt}} \in [{\lambda _{min}},{\lambda _{max}}]$ by referring to the meeting point of the strain energy curves, calculated for the eight different DOF, with the reference solution (indicated by the black solid line), where ${\lambda _{min}} \approx 0.8$ and ${\lambda _{max}} \approx 1$. To verify the feasibility of the proposed method, the intermediate value $\lambda =0.9$ within the optimal range $[0.8,1]$ was selected as the optimal parameter. Calculations were then performed on twist drills made of three different materials to confirm that the obtained approximate solutions for displacement, stress, and strain were optimal.

**Table 11:** Different grid types of twist drills and related information.

Model	Type	Node	Element	DOF
Model 1	Mesh 1	1112	3882	3336
Model 2	Mesh 2	1472	5328	4416
Model 3	Mesh 3	1751	6425	5253
Model 4	Mesh 4	1904	7089	5712
Model 5	Mesh 5	2061	7771	6183
Model 6	Mesh 6	2508	9994	7524
Model 7	Mesh 7	3330	13,752	9990
Model 8	Mesh 8	4223	17,979	12,669
Reference	Mesh 9	40,590	202,048	121,770

**Table 12:** Mesh quality inspection of twist drills.

Type	Aspect ratio (Max)	Warpage (Max)	Skew (Max)	Jacobian (Min)
Mesh 1	5.24(2 of 3882)	0	0.88	1
Mesh 2	3.92	0	0.75	1
Mesh 3	4.03	0	0.75	1
Mesh 4	3.91	0	0.75	1
Mesh 5	4.11	0	0.75	1
Mesh 6	4.02	0	0.74	1
Mesh 7	3.67	0	0.74	1
Mesh 8	3.86	0	0.75	1
Mesh 9	4.23	0	0.79	1

**Fig. 21:**
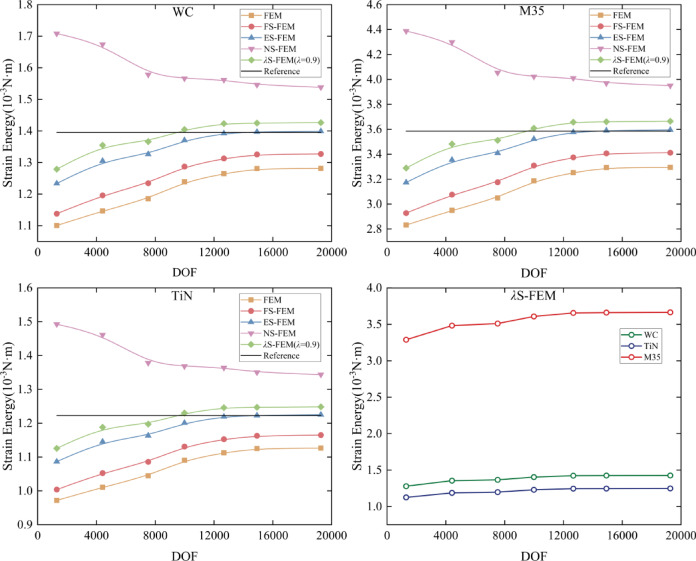
The strain energy convergence curve of three kinds of twist drills.

To verify convergence, the convergence curves of the strain energies for the three twist drills, obtained by the five methods, are presented in Fig. 21. As depicted in the figure, *λ*S-FEM ($\lambda =0.9$) demonstrates superior accuracy compared to FEM and S-FEM when utilizing the same tetrahedral mesh. Additionally, the overall strain energy of M35 material is higher than that of the other two materials, indicating that the deformation of M35 is greater than that of the other materials.

To assess the accuracy and reliability of the method, strain energy errors were calculated for each approach. The strain energy error equation can be expressed as:

26 $${e_e}=\sqrt {\frac{{\left| {{E^{ref}} - {E^{num}}} \right|}}{{{E^{ref}}}}}$$

${E^{ref}}$ represents the reference solution and ${E^{num}}$ represents the computational solution.

**Fig. 22:**
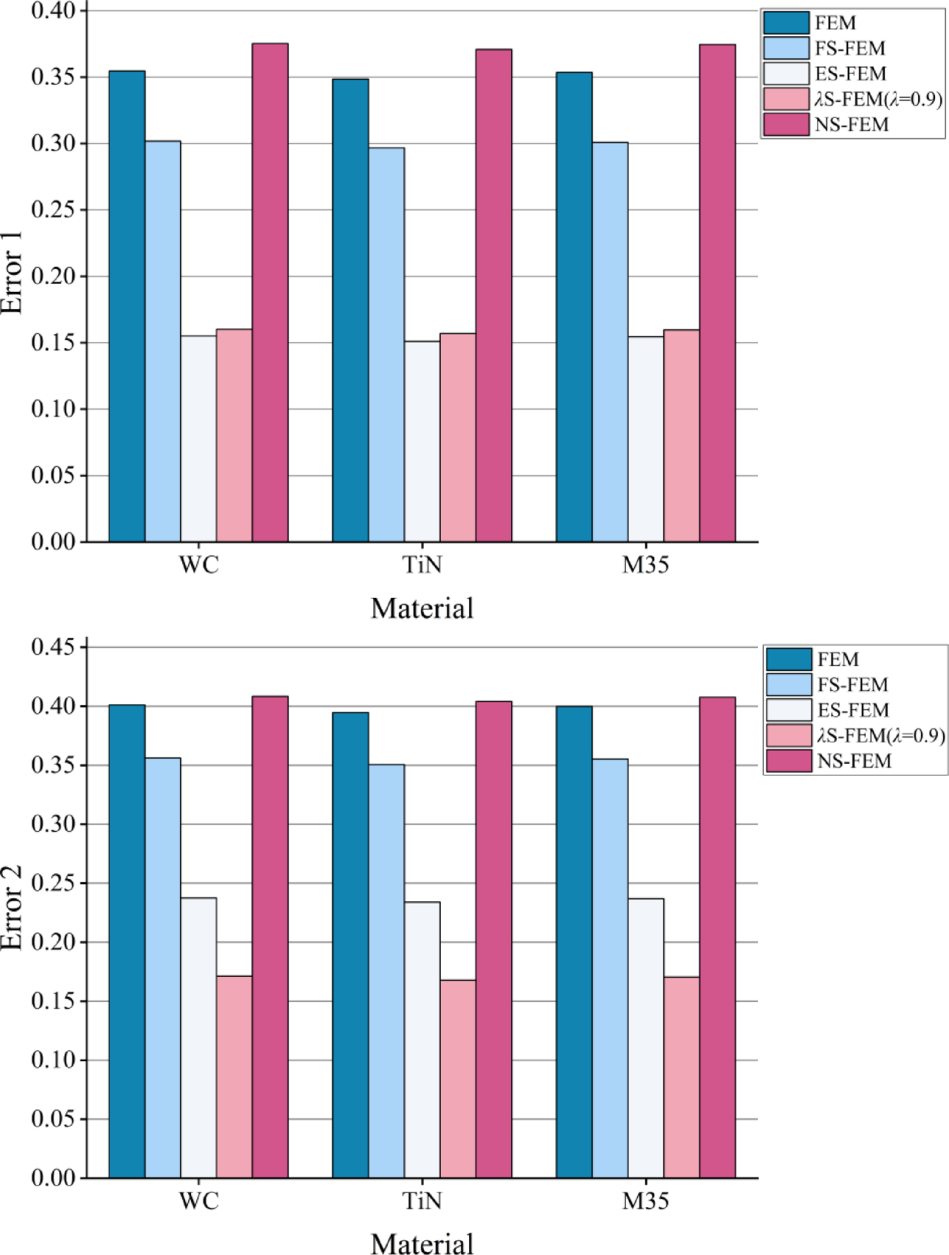
The strain energy error of three kinds of twist drills.

The strain energy errors are presented in Fig. 22, where Error 1 denotes the error associated with the overall degree of freedom, while Error 2 refers to the error for lower degrees of freedom (less than 10,000). The results indicate that the errors in the overall degrees of freedom are ranked from smallest to largest as follows: ES-FEM, *λ*S-FEM ($\lambda =0.9$), FS-FEM, FEM, and NS-FEM. Among them, *λ*S-FEM ($\lambda =0.9$) ranks second only to ES-FEM and has the smallest strain energy error at low degrees of freedom, with values of 0.171187, 0.167804, and 0.170565, which are only 42% of those for NS-FEM.

### Numerical results analysis of twist drill

This section presents detailed numerical results of the *λ*S-FEM to demonstrate its accuracy in solving the twist drill mechanics problem. These results are compared with those from the FEM and S-FEM models, focusing on displacement and stress.

**Fig. 23:**
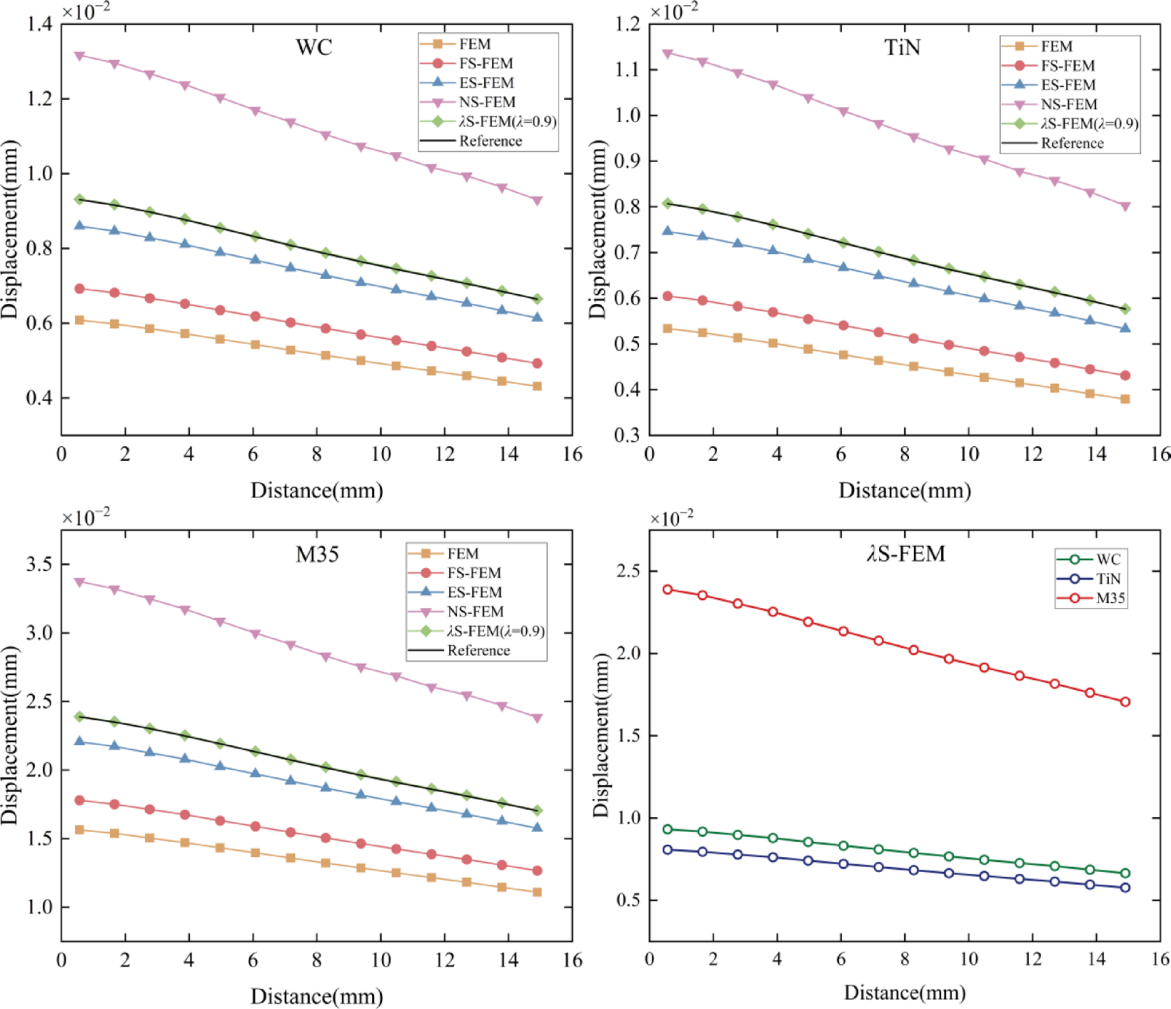
The displacement distribution of three kinds of twist drills along the AB helix.

To perform the small deformation analysis of the twist drill, helix AB in Fig. 19c is selected to examine the displacement of 14 nodes along the helix when the degree of freedom is 7524, as shown in Fig. 23. The figure illustrates that the displacements decrease progressively. The part of the drill near the cutting edge, which is in direct contact with the workpiece, experiences significant deformation, while the part farther from the cutting edge (e.g., the shank), subjected to relatively smaller forces, undergoes minimal deformation. The *λ*S-FEM ($\lambda =0.9$) combines the characteristics of the upper-bound solution of NS-FEM and the lower-bound solution of ES-FEM to provide a displacement solution that closely matches the reference solution.

**Fig. 24:**
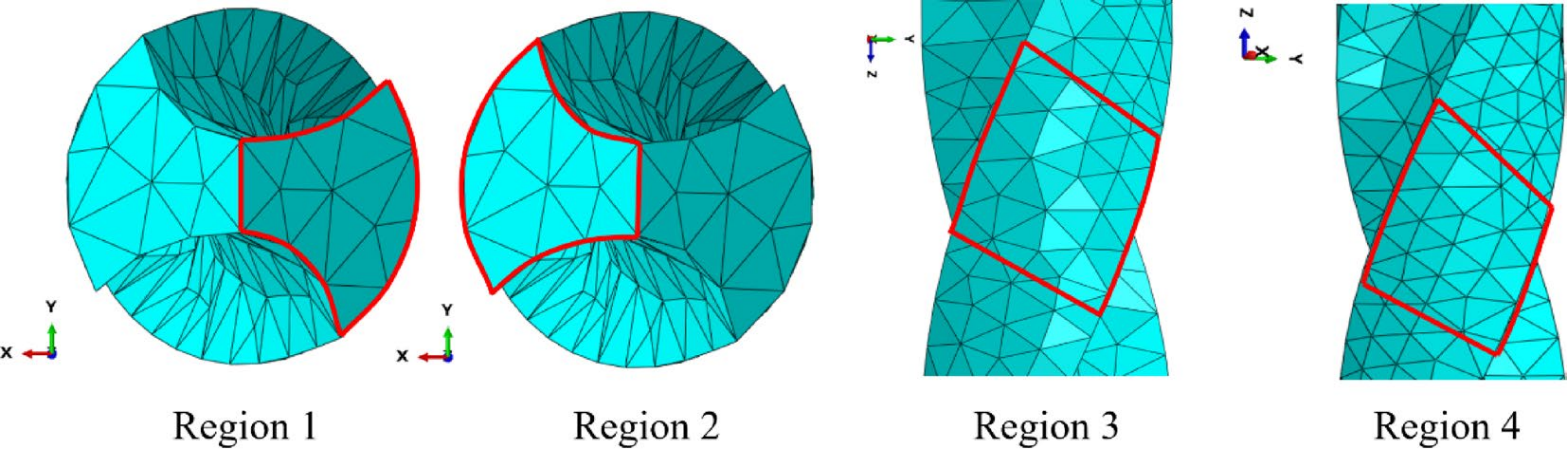
The four sub-regions of the twist drill model.

To confirm the correctness and stability of the *λ*S-FEM model, the displacement errors of various methods were analyzed and compared. The displacement error of the S-FEM model is defined as:

27 $${e_d}=\frac{{\left| {{u_i} - {{\bar {u}}_i}} \right|}}{{{u_i}}} \times 100\%$$

where ${u_i}$ denotes the reference solution and ${\bar {u}_i}$ denotes the computational solution. The displacement errors for the 14 nodes are calculated according to Fig. 23 and Eq. (27), as shown in Table 13. It is evident that *λ*S-FEM ($\lambda =0.9$) achieves the smallest errors of 0.0019, 0.001674, and 0.001873, respectively, are approximately 2.5% of the errors observed in ES-FEM. Additionally, NS-FEM exhibits the largest error. To ensure a comprehensive and systematic assessment, displacement errors for the four selected regions (as shown in Fig. 24), along with the global errors for these regions, were computed, and the corresponding results are presented in Table 14.

**Table 13:** Displacement errors of the 14 nodes along helix AB.

Material	WC	TiN	M35
FEM	0.347615	0.339723	0.346118
FS-FEM	0.256029	0.250437	0.254951
ES-FEM	0.075045	0.074240	0.074886
*λ*S-FEM ($\lambda =0.9$)	0.001900	0.001674	0.001873
NS-FEM	0.408447	0.401852	0.407164

**Table 14:** Global displacement error of three kinds of twist drills.

Material	Region	FEM	FS-FEM	ES-FEM	*λ*S-FEM (*λ* = 0.9)	NS-FEM
WC	Region 1	0.2753	0.2051	0.0652	0.0127	0.3244
	Region 2	0.2910	0.2170	0.0688	0.0126	0.3496
	Region 3	0.3410	0.2506	0.0717	0.0048	0.4065
	Region 4	0.3552	0.2602	0.0730	0.0061	0.4222
	Global error	0.3242	0.2391	0.0704	0.0081	0.3859
TiN	Region 1	0.2683	0.2001	0.0643	0.0125	0.3183
	Region 2	0.2825	0.2109	0.0676	0.0125	0.3420
	Region 3	0.3332	0.2452	0.0711	0.0045	0.3998
	Region 4	0.3460	0.2538	0.0722	0.0057	0.4142
	Global error	0.3159	0.2333	0.0695	0.0078	0.3787
M35	Region 1	0.2741	0.2042	0.0650	0.0127	0.3232
	Region 2	0.2894	0.2158	0.0685	0.0126	0.3482
	Region 3	0.3395	0.2496	0.0716	0.0048	0.4052
	Region 4	0.3534	0.2590	0.0729	0.0061	0.4207
	Global error	0.3226	0.2380	0.0702	0.0081	0.3845

**Fig. 25:**
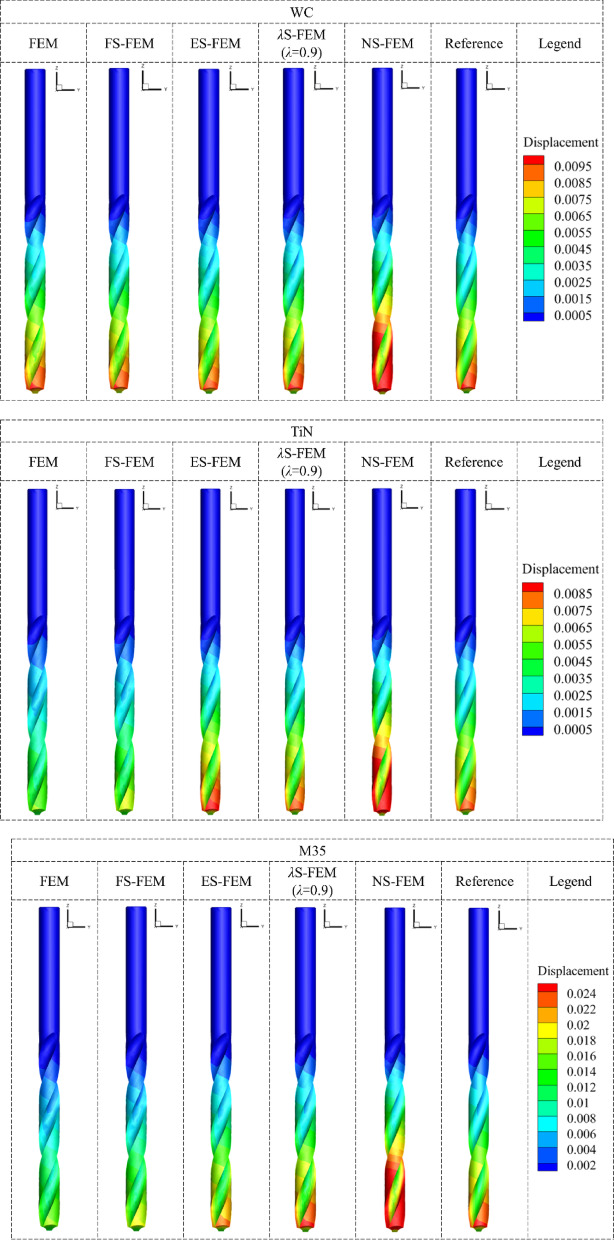
Comparison of surface displacement results of twist drills (mm).

The results of the surface displacement field for the twist drill with three materials, WC, TiN and M35, are shown in Fig. 25. Orange and red areas in the diagram represent regions where the numerical simulation results indicate greater displacement. The drill bit experiences the most deformation, with the corresponding deformations for WC and TiN drills being 0.01 mm and 0.00868 mm, respectively. In comparison, the maximum deformation of the M35 drill is 0.0257 mm, which is 2.57 and 2.96 times larger than that of the WC and TiN drills, respectively. Consequently, after extended machining, the durability performance of M35 is lower than that of WC and TiN. The displacement distribution of *λ*S-FEM ($\lambda =0.9$) closely resembles the key region of the reference solution, indicating that the *λ*S-FEM ($\lambda =0.9$) displacement solution aligns well with the reference solution. The displacement results for three nodes of the twist drill with three materials, based on three grid densities and calculated using different analysis methods, are shown in Table 15. The table also presents the displacement results of *λ*S-FEM at three different values of $\lambda$. The *λ*S-FEM weighting method for ES-FEM and NS-FEM generally produces displacement results that lie between the bounds calculated by ES-FEM and NS-FEM. When taken within the optimal range, *λ*S-FEM consistently provides the most accurate approximate solution.

**Table 15:** The displacement results of three nodes for three mesh densities (mm).

Material	Point	Node	NS-FEM	ES-FEM	*λ*S-FEM			Reference
			$\times {e^{ - 03}}$	$\times {e^{ - 03}}$	$\lambda =0.85$ $\times {e^{ - 03}}$	$\lambda =0.9$ $\times {e^{ - 03}}$	$\lambda =0.95$ $\times {e^{ - 03}}$	$\times {e^{ - 03}}$
WC	1	1472	7.64	5.41	5.85	5.69	5.55	5.84
		2508	6.95	5.51	5.84	5.73	5.62	
		3330	6.82	5.75	6.02	5.93	5.84	
	2	1472	0.81	0.57	0.62	0.60	0.59	0.60
		2508	0.74	0.58	0.62	0.61	0.59	
		3330	0.73	0.59	0.62	0.61	0.60	
	3	1472	0.37	0.26	0.28	0.27	0.27	0.27
		2508	0.33	0.26	0.28	0.27	0.27	
		3330	0.34	0.27	0.28	0.28	0.27	
TiN	1	1472	6.65	4.74	5.11	4.98	4.85	5.11
		2508	6.05	4.82	5.11	5.01	4.91	
		3330	5.94	5.03	5.26	5.18	5.11	
	2	1472	0.72	0.50	0.55	0.53	0.52	0.53
		2508	0.65	0.51	0.55	0.53	0.52	
		3330	0.64	0.52	0.55	0.54	0.53	
	3	1472	0.31	0.23	0.24	0.24	0.23	0.24
		2508	0.28	0.23	0.24	0.24	0.24	
		3330	0.29	0.23	0.25	0.24	0.24	
M35	1	1472	19.61	13.90	15.02	14.62	14.25	15.00
		2508	17.85	14.15	15.01	14.71	14.43	
		3330	17.50	14.76	15.47	15.24	15.00	
	2	1472	2.09	1.46	1.60	1.55	1.51	1.54
		2508	1.90	1.49	1.59	1.56	1.52	
		3330	1.88	1.51	1.60	1.57	1.54	
	3	1472	0.93	0.67	0.72	0.70	0.68	0.70
		2508	0.84	0.68	0.72	0.70	0.69	
		3330	0.87	0.69	0.72	0.71	0.70	

**Fig. 26:**
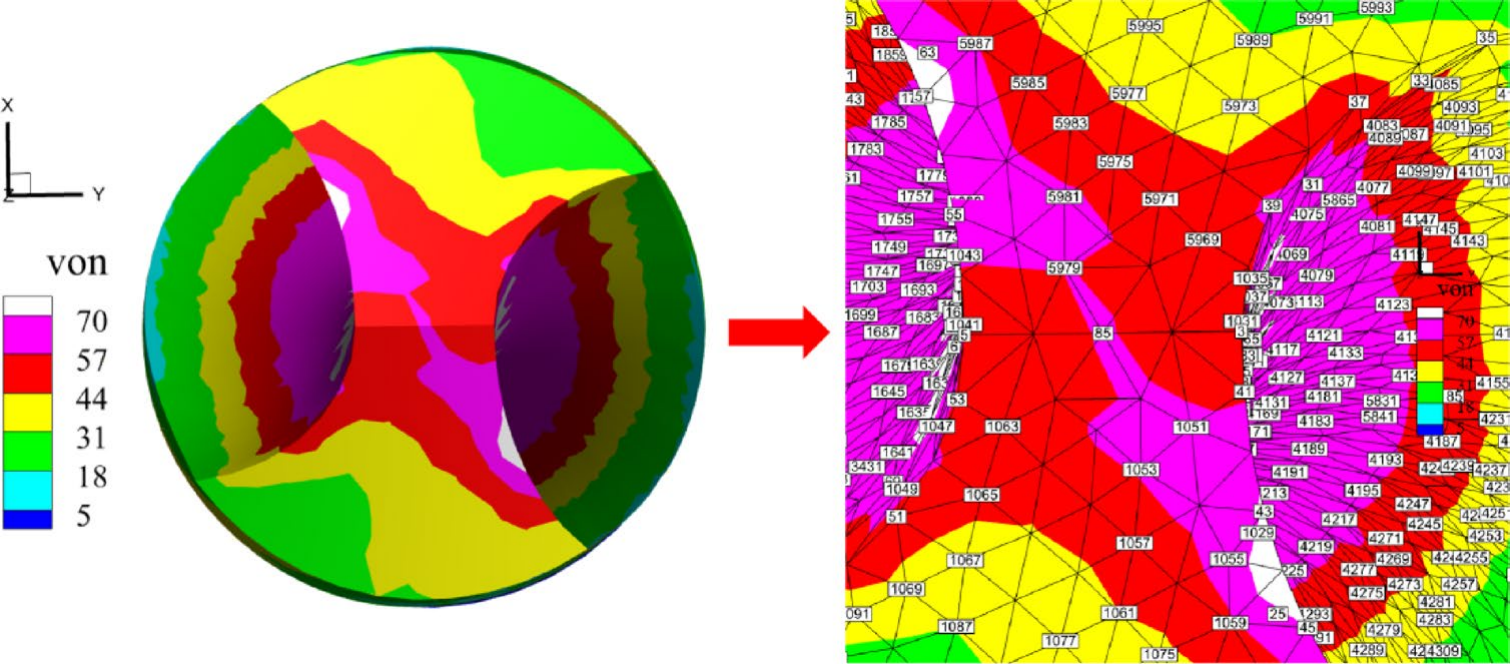
Maximum stress area of twist drills.

Von Mises stress is widely utilized in engineering, especially in mechanical design and structural analysis, to assess material strength under various stress conditions. It is derived from the stress components using the following formula:

28 $${\sigma _v}=\sqrt {\frac{1}{2}\left[ {{{\left( {{\sigma _{11}} - {\sigma _{22}}} \right)}^2}+{{\left( {{\sigma _{22}} - {\sigma _{33}}} \right)}^2}+{{\left( {{\sigma _{33}} - {\sigma _{11}}} \right)}^2}+6\left( {\sigma _{{12}}^{2}+\sigma _{{23}}^{2}+\sigma _{{13}}^{2}} \right)} \right]}$$

where, ${\sigma _{11}}, {\sigma _{22}}, {\sigma _{33}}$ represent the positive stresses in three directions and ${\sigma _{12}}, {\sigma _{23}}, {\sigma _{13}}$ represent the shear stresses. As shown in Table 16, the stress values at ten random nodes on the twist drill are compared. These results indicate that the stress solution provided by *λ*S-FEM ($\lambda =0.9$) is more accurate and effective for analyzing the mechanical problems of twist drills. The region of maximum stress is illustrated in Fig. 26, which clearly indicates that the peak stress of the twist drill occurs near the cutting edges. The corresponding maximum stress and strain values are presented in Table 17.

**Table 16:** The stress results of three kinds of twist drills (N/mm^2^).

Point		1	2	3	4	5	6	7	8	9	10
WC	FEM	34.03	34.13	33.65	33.92	31.68	16.79	17.54	25.44	1.386	1.368
	FS-FEM	36.31	36.50	32.69	33.18	30.99	17.08	17.80	24.20	1.454	1.429
	ES-FEM	39.40	39.63	31.02	32.02	29.82	17.41	18.13	22.48	1.578	1.533
	*λ*S-FEM ($\lambda =0.9$)	40.17	40.35	30.21	31.48	29.12	18.12	18.90	20.95	1.652	1.596
	NS-FEM	59.21	64.19	22.24	57.25	24.33	26.39	28.86	15.87	2.176	1.929
	Reference	42.22	42.46	29.09	29.21	28.15	21.28	21.51	20.26	1.649	1.625
TiN	FEM	34.24	34.35	33.43	33.80	31.71	17.09	17.84	25.57	1.285	1.256
	FS-FEM	36.38	36.58	32.49	33.06	31.11	17.50	18.17	24.42	1.346	1.309
	ES-FEM	39.37	39.59	30.92	31.94	29.99	17.94	18.58	22.84	1.463	1.405
	*λ*S-FEM ($\lambda =0.9$)	40.11	40.27	30.13	31.40	29.28	18.66	19.35	21.33	1.530	1.461
	NS-FEM	59.37	64.56	22.50	27.08	24.40	27.31	29.81	10.56	2.038	1.732
	Reference	41.78	42.11	29.04	29.09	28.37	21.58	21.77	20.63	1.502	1.478
M35	FEM	34.08	34.18	33.61	25.46	31.68	16.86	17.61	33.90	1.367	1.346
	FS-FEM	36.33	36.53	32.65	33.15	31.01	17.17	17.88	24.23	1.433	1.405
	ES-FEM	39.40	39.63	31.00	32.00	29.85	17.52	18.22	22.54	1.555	1.508
	*λ*S-FEM ($\lambda =0.9$)	40.17	40.34	30.19	31.46	29.15	18.24	19.00	21.01	1.628	1.569
	NS-FEM	59.25	64.26	22.28	27.17	24.34	26.58	29.06	10.35	2.149	1.891
	Reference	42.14	42.39	29.07	29.18	28.18	21.36	21.58	20.31	1.620	1.596

**Table 17:** Maximum stress and strain results of three kinds of twist drills (N/mm^2^).

Material	Method	Maximum stress (Von)	Maximum strain (ɛ_xx_)
WC	FEM	51.89	3.80E-05
	FS-FEM	54.02	3.91E-05
	ES-FEM	57.59	4.22E-05
	*λ*S-FEM ($\lambda =0.9$)	60.81	4.54E-05
	NS-FEM	110.38	8.28E-05
	Reference	82.73	5.31E-05
TiN	FEM	51.92	2.85E-05
	FS-FEM	54.00	2.93E-05
	ES-FEM	57.44	3.33E-05
	*λ*S-FEM ($\lambda =0.9$)	60.63	3.61E-05
	NS-FEM	110.27	6.91E-05
	Reference	82.72	4.42E-05
M35	FEM	51.90	9.52E-05
	FS-FEM	54.02	9.81E-05
	ES-FEM	57.57	1.07E-04
	*λ*S-FEM ($\lambda =0.9$)	60.79	1.15E-04
	NS-FEM	110.38	2.11E-04
	Reference	82.74	1.35E-04

**Fig. 27:**
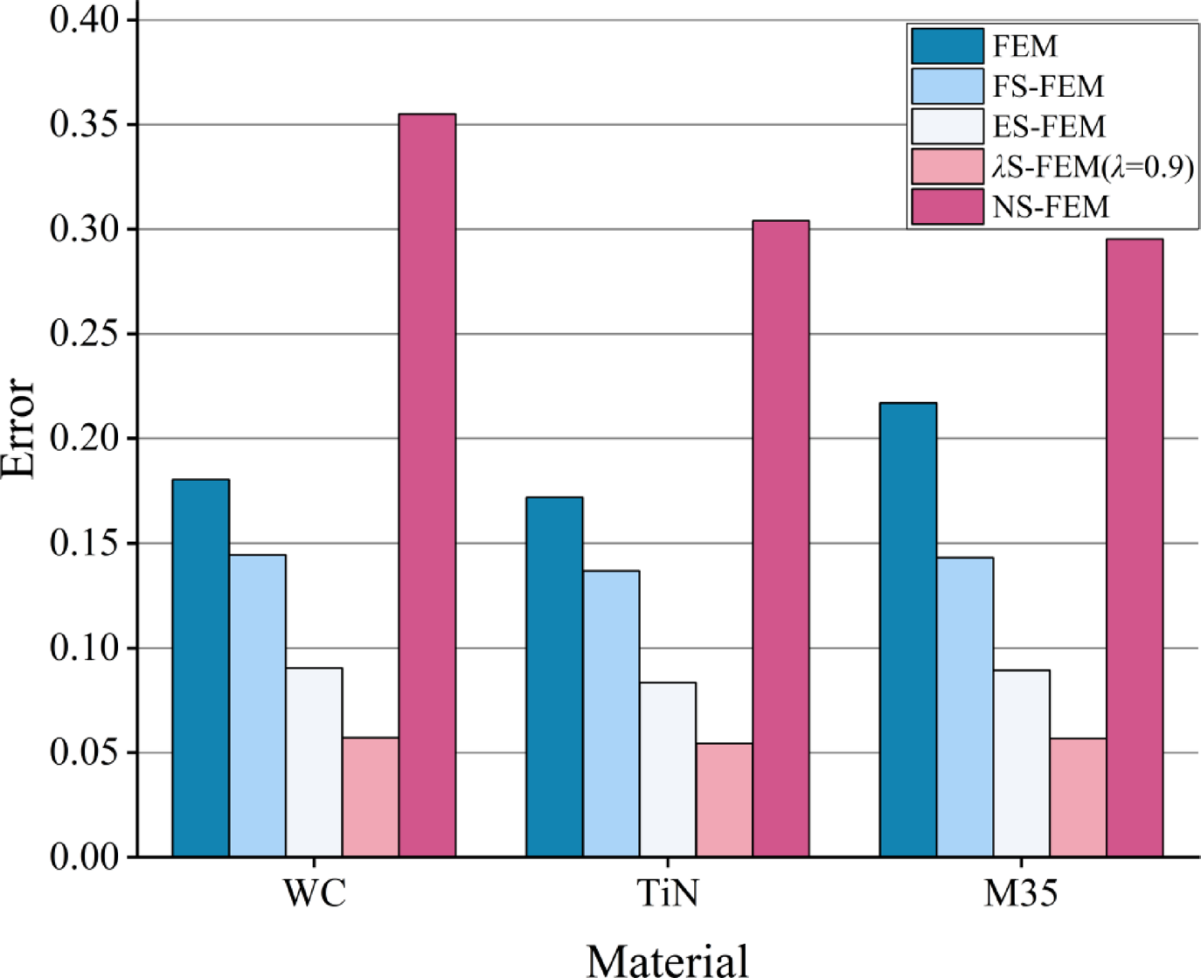
The stress error of three kinds of twist drills.

The stress error is defined in the S-FEM model:

29 $${e_v}=\frac{{\left| {{\sigma _i} - {{\bar {\sigma }}_i}} \right|}}{{{\sigma _i}}} \times 100\%$$

where ${\sigma _i}$ represents the reference stress of node *i* and ${\bar {\sigma }_i}$ denotes the numerical stress of node *i*. The average stress errors for the ten nodes, calculated using Table 16 and Eq. (29), are presented in Fig. 27. *λ*S-FEM ($\lambda =0.9$) has the smallest errors of 0.057128, 0.054333, and 0.056749(approximately 30% of FEM), respectively, followed by ES-FEM (40-50% of FEM). Therefore, the accuracy of static analysis for twist drills using *λ*S-FEM with appropriate parameters $\lambda$ is superior to that of the FEM and S-FEM methods.

**Fig. 28:**
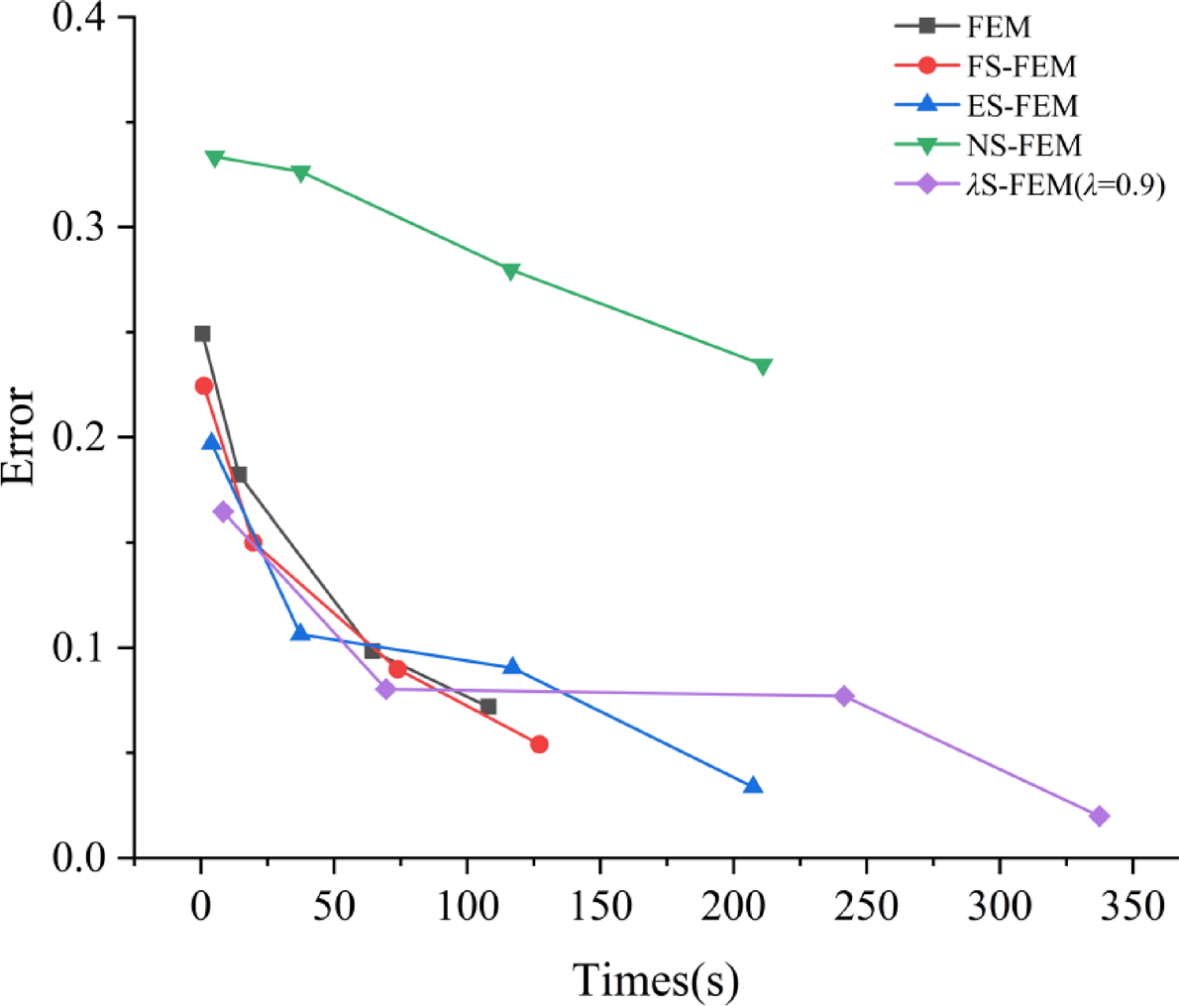
Efficiency curves of different finite element methods.

To evaluate the computational time of *λ*S-FEM, four different degrees of freedom (1296, 7524, 14919, 19269) ranging from small to large, are selected in this study, and the computational efficiencies of various methods are compared. Efficiency curves are plotted with stress error on the vertical axis and total computation time on the horizontal axis, as shown in Fig. 28. Since *λ*S-FEM combines NS-FEM and ES-FEM, it requires cyclic traversal of all edges and nodes, resulting in longer analysis times. However, *λ*S-FEM demonstrates high accuracy within an acceptable time frame, and both analysis time and accuracy improve as the degrees of freedom increase.

## Conclusion

This paper introduces the *λ*S-FEM method and its application to the mechanical analysis of 3D twist drills. The approach involves constructing a smoothing model using a domain generated by a coupled smoothing technique, calculating strain energy under varying DOF, and presenting the results as a curve. The optimal range for the parameter $\lambda$ is determined from the intersection of the curve with the reference solution. This range enables the selection of an appropriate $\lambda$ value for mechanical analysis. Theoretical and numerical investigations demonstrate that *λ*S-FEM offers high accuracy. The findings are summarized as follows:

1. Parameter control

*λ*S-FEM regulates the contributions of ES-FEM and NS-FEM through the parameter $\lambda$. When $\lambda =0$, *λ*S-FEM corresponds to NS-FEM, and when $\lambda =1$, it aligns with ES-FEM, providing a seamless transition between these two methods.

(2) Enhanced accuracy

In solid mechanics, *λ*S-FEM mitigates the “too soft” property of NS-FEM, achieving higher accuracy in the displacement and stress fields for static problems. Results from *λ*S-FEM surpass those obtained using FEM and S-FEM on the same mesh.

(3) Optimal parameter range

For straight shank twist drills, the optimal range for $\lambda$ is identified. *λ*S-FEM with an optimized $\lambda$ reduced the strain energy error of twist drills by 57–58% compared to NS-FEM, showcasing its suitability for mechanical analysis.

(4) Material stress accuracy

*λ*S-FEM computations achieve superior accuracy over FEM and S-FEM for the same degree of freedom. Compared to ES-FEM, *λ*S-FEM improves the stress accuracy for twist drills made from WC, TiN, and M35 materials by 58.3%, 53.6%, and 57.4%, respectively.

(5) Drilling performance

During drilling of stainless steel 304, WC and TiN twist drills exhibit smaller displacement changes and lower overall deformation, enhancing durability and machining efficiency. In contrast, M35 twist drills show greater deformation, leading to higher wear, reduced durability, and decreased machining efficiency.

## Data Availability

The data generated in this study are available from the corresponding author upon reasonable requests.
